# On the establishment of reference values of clouds of electromyography interference pattern by linear regression method and percentile method and comparison of sensitivity and specificity of both methods

**DOI:** 10.3389/fneur.2022.917308

**Published:** 2022-09-01

**Authors:** Dan Wang, Lei Liu, Ruohong Xue, Zhongming Li, Yuqi Gao, Ting Wang, Yanfang Kang, Jingjing Wang, Qiuye Yin, Najuan Li, Yanbing Han

**Affiliations:** ^1^Department of Neurology, First Affiliated Hospital of Kunming Medical University, Kunming, China; ^2^Department of Human Anatomy and Histoembryology, School of Basic Medical Sciences, Kunming Medical University, Kunming, China; ^3^School of Basic Medical Sciences, Kunming Medical University, Kunming, China; ^4^Department of Clinical Pharmacy, First Affiliated Hospital of Kunming Medical University, Kunming, China; ^5^The First Clinical College of Kunming Medical University, Kunming, China

**Keywords:** electromyography, linear regression clouds, percentile clouds, reference values, sensitivity, specificity

## Abstract

**Objective:**

Turn-amplitude clouds were widely used in automatic electromyography (EMG) interference pattern analysis. Earlier works employed the intercept ± 2SD (standard deviation) of the linear regression equation as the upper and lower boundaries of the clouds. The goal of this study was to employ the linear regression method and percentile method to calculate the reference value of turn-amplitude clouds, identify the determining criteria in accordance with the receiver operator characteristic curve (ROC), and analyze the sensitivity and specificity of the linear regression cloud, percentile cloud, and quantitative assessment of the motor unit potential (QMUP).

**Methods:**

First, we explore what factors affect the number of turns per second and the mean amplitude. Then, their logarithms were taken for the normal test. All muscle data were used to calculate the reference values of percentile clouds. However, the reference values of the linear regression clouds were obtained for the muscles with a bivariate normal distribution, homogeneous variances and a linear correlation. We calculated the prediction interval with the standard errors of the intercept and slope of the linear regression equation, which can determine the upper and lower boundaries of the linear regression clouds. Furthermore, we obtained ROCs of these clouds, which were used as the determining criteria to determine the optimum cut-off values. Finally, our study analyzed the sensitivity and specificity of the linear regression cloud, percentile cloud, and QMUP.

**Results:**

We here presented the reference values and ROCs of the linear regression clouds and percentile clouds. We suggest the determining criteria be based on ROCs. The areas under the curve (AUC) of both clouds are larger than 0.8, revealing that they have significant diagnostic value. Our results display that the specificities of the linear regression clouds, percentile clouds, and QMUP are almost identical to each other, whereas the sensitivity of percentile cloud is higher than those of QMUP and linear regression clouds.

**Conclusion:**

According to ROCs, the researchers determine the determining criteria of the linear regression clouds and percentile clouds. Our findings suggest that the percentile clouds possess a wide application range and significant diagnostic value, therefore it may be the optimum for automatic EMG interference pattern analysis.

## Introduction

Needle electromyography (EMG) plays a key role in medical electrodiagnostics. By observing insertional electrical activity, spontaneous electrical activity, quantitative assessment of the motor unit potential (QMUP), and visual assessment of the interference pattern, we analyzed electrical activity of skeletal muscle to identify neuropathic or myopathic lesion. In particular, QMUP reveals the electrical activity of the motor units through analyzing the motor units activated by small force contraction of muscle. By contrast, *via* the visual assessment, we observe the interference pattern formed by the recruitment of various motor units during maximal voluntary muscle contraction. This assessment can evaluate a wider range of motor units. There is no difference in the sensitivity and specificity of QMUP and the visual assessment (1).

In 1941, Buchthal and Clemmensen (2) proposed QMUP. This method was suitable to the single motor unit potential (MUP) of muscle contraction force that was only about 4% of the maximum random contraction force. Therefore, the QMUP only reflects the state of the motor unit activated by small force contraction. Also, the collection of motor units and measurement of its duration depended on the subjective judgment of EMG examiners (2, 3). To improve inspection efficiency and objective judgment, there was an urgent need to develop a simple inspection method to implement easily. Currently, researchers have developed a variety of automatic analysis techniques of electromyography (EMG) interference pattern, including the turn-amplitude clouds (4–7), the number of small segments (NSS)-activity clouds analysis (8–10), and quantitative evaluation of interference patterns on EMG in neuropathy (11). It is worth mentioning that turn-amplitude clouds were widely used for these methods.

In 1983, Stålberg et al. first employed the mean amplitude and the number of turns per second at various levels of muscle contraction force to establish clouds in the Plane Rectangular Coordinate System. The upper and lower boundaries of the clouds were determined by the ±2 standard deviation (SD) of the regression line in the linear regression equation, which yields the 95% prediction interval for the mean amplitude at a specific number of turns per second (4). Nandedkar et al. also used the identical method to determine the upper and lower boundaries of the clouds (5). To determine the upper and lower boundaries of the clouds, other turn-amplitude clouds investigations (6, 7) usually employ intercept ± 2 SD of the linear regression equation. Jabre et al. (6) found that the linear regression was only suitable for muscle contraction from small to moderate force, when the contraction force was increased from 50% of the maximum contraction force, there was no linear correlation between the number of turns per second and the mean amplitude. In a word, the upper and lower borders of the clouds created using the linear regression approach in the study mentioned above contain the confidence interval of the overall mean of amplitude, and they did not provide the evidence of judgment standard of the clouds. In this study, the intercept and slope of the linear regression equation were used to determine the upper and the lower boundaries of the clouds that contains the prediction interval of the mean amplitude corresponding to the number of turns per second, rather than the confidence interval.

There are restrictions on the application of the linear regression clouds, namely, it can be applied only when there is linear correlation between the number of turns per second and the mean amplitude with normal distribution and the linear regression equation can be established. However, some muscle data do not fit the condition listed above, which makes it impossible to develop the linear regression clouds. Therefore, we found the percentile clouds have the following advantages over the linear regression clouds: first, it does not require normal distribution of data and is appropriate for all muscles; second, the data collected from the slightest contraction force to the strongest contraction force can reflect the information of more motor units more comprehensively; third, there was no significant statistical difference in the specificity of the linear regression clouds, percentile clouds, and QMUP, but the sensitivity and Youden's index of the percentile clouds were the highest, indicating that it has significant diagnostic value.

This study analyzes the factors that affect the number of turns per second and the mean amplitude of electromyographic interference patterns. By the linear regression equation, we calculated the prediction interval of the mean amplitude with the given the number of turns per second, which can determine the upper and lower boundaries of the linear regression clouds. To achieve this goal, the linear regression method and the percentile method are adopted to establish reference values of EMG clouds for the eight muscles commonly used in EMG examination. With ROCs as the judging criteria of two clouds, the sensitivity and specificity of the linear regression clouds, the percentile clouds, and QMUP are analyzed.

## Methods

### Healthy subjects and patients information

This study was approved by the Ethics Committee of the First Affiliated Hospital of Kunming Medical University (Approval No. 2020.1.13). All subjects were informed and signed informed consents. From February 2020 to July 2020, healthy subjects were recruited in the First Affiliated Hospital of Kunming Medical University, and the data were collected to establish reference values of clouds. From August 2020 to April 2021, the patients who were hospitalized in the First Affiliated Hospital of Kunming Medical University were informed and received an EMG examination. Then, the collected data were used to analyze the sensitivity and specificity of QMUP, the linear regression clouds, and percentile clouds.

The inclusion criteria for healthy subjects are (1) ≥18 years old; (2) healthy people; (3) no neurological symptoms or signs. The exclusion criteria for healthy subjects are (1) abnormal blood coagulation function; (2) suffering from systemic disease; (3) muscle strength to measure manually below level 5. The inclusion criteria for inpatients are (1) ≥18 years old; (2) accepting nerve conduction study and needle EMG. The exclusion criteria for inpatients are (1) unclear diagnosis; (2) neuromuscular junction disease; (3) abnormal blood coagulation function; (4) suffering from systemic diseases.

Two physicians with EMG examination qualifications collected data and determined the measurement duration of motor unit potential (MUP). The results of EMG diagnosis were performed by an attending physician. The examiners have received strict professional training and did not know the patient's final clinical diagnosis.

The data collection of healthy volunteers involved inquiry of medical history and comprehensive nervous system examination. First, the nerve conduction studies were performed. Then, the needle EMG examinations were carried out to evaluate the insertional activity, spontaneous activity, QMUP, and the interference pattern of each muscle. Normal muscles of healthy volunteers were included in the healthy group.

The data collection of inpatients involved inquiry of medical history and neurological physical examination. According to the results of the nerve conduction study, insertional activity, spontaneous activity, QMUP, and the interference pattern of EMG, the muscles of inpatients were classified into neuropathic lesion group, myopathic lesion group, or healthy muscles group, respectively. The data from −20% to +20% of the average duration of the normal MUP developed in our research unit was considered as normal values (12).

### Recording and analysis

The data were collected using EDX20.0 EMG machine from Nicolet, USA. The concentric needle electrodes are from Technomed Europe, Netherlands, with a length of 37 mm, a diameter of 0.45 mm, and a recording area of 0.068 square millimeter. The parameter settings were scanning speed of 20 ms/div, gain of 1 mV/div, filter bandwidth from 20 to 10 kHz, data sampling rate ≥90 kHz/channel, ADC capacity of 24 bits, noise suppression ≤1 μV, common -mode rejection ratio ≥120 dB, notch filter of 50 Hz, and signal acquisition time of 400 ms.

Data collection of electromyographic interference pattern: For each subject, the data collection was performed on muscles of different name, including sternocleidomastoid, deltoid, biceps brachii, extensor digitorum communis, abductor digiti minimi, vastus medialis, tibialis anterior, and gastrocnemius. Four parts were randomly selected from each muscle. The examinees were instructed to contract the muscles against the examiner, starting from a small contraction force to the maximum contraction force (see Supplementary material). Under different contraction forces, five data records were collected for each part of the muscle, and the data collection interval was 1 min In this process, a total of 20 data records were collected for each muscle, and each data record contained the number of turns per second and its corresponding mean amplitude. The data records of the muscles of same name collected from different subjects were classified into the same group, and the dominant and non-dominant limb muscles were distinguished.

Since most of the number of turns per second and mean amplitude of the collected data were not normally distributed, their logarithms based on 10 were taken to test the normality. The use of *t*-test or one-way analysis of variance or Wilcoxon rank sum test or Kruskal-Wallis *H* test depended on whether the lg (number of turns per second) and lg (mean amplitude) were normally distributed and the variances were homogeneous. Also, lg (the number of turns per second) and lg (the mean amplitude) of the dominant and non-dominant limb muscles groups, groups of different genders, and groups of different age were compared to find whether there are the differences of statistical significance. If there were no differences, the data of the muscles of same names were combined to establish a reference value; otherwise, the reference values were established separately.

### Two methods to develop the clouds

**Figure 1:**
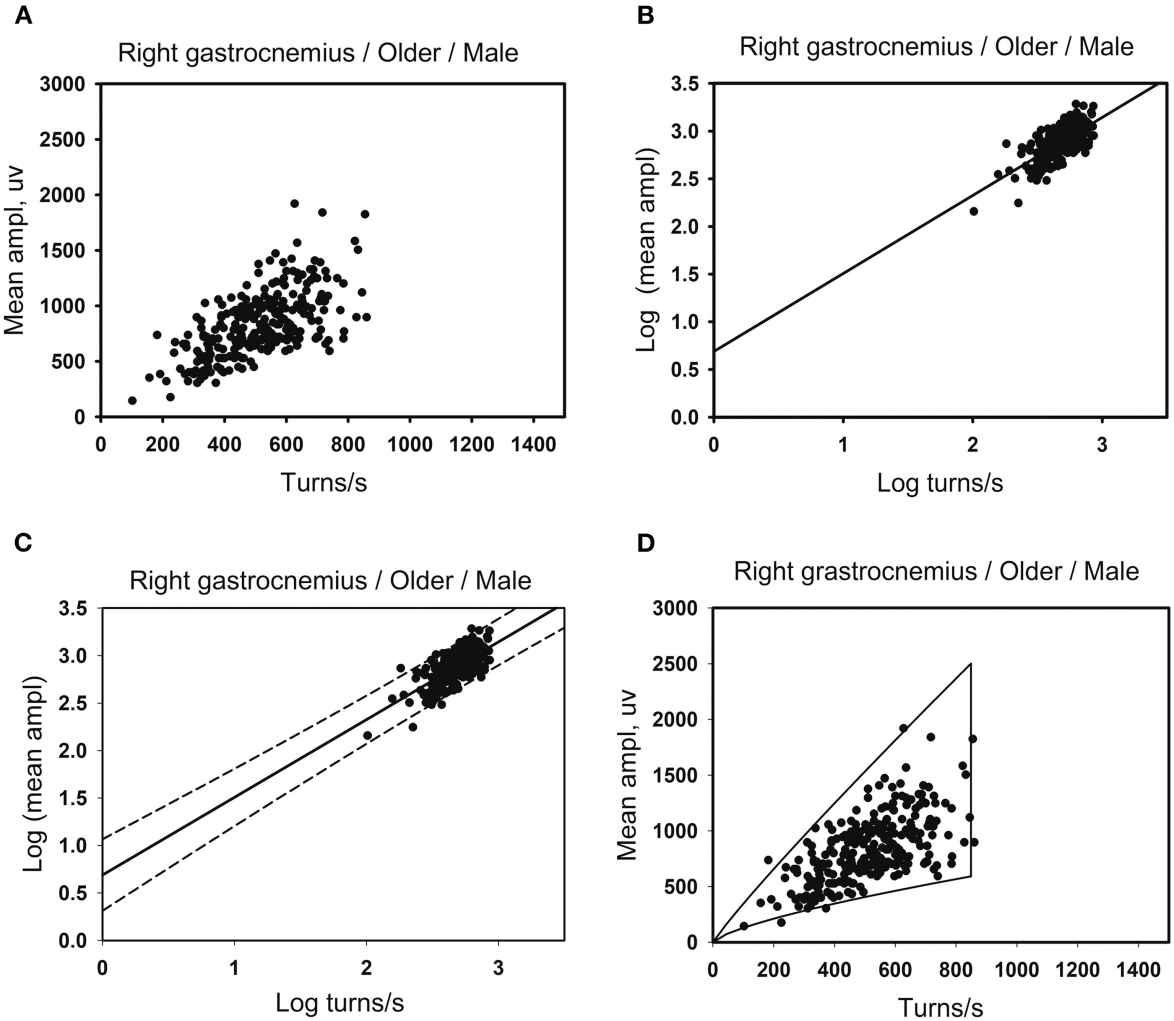
Developing method of the linear regression clouds. **(A)** There are 260 data points for the right gastrocnemius muscles in the older men. Scatter plots are made using the number of turns per second and the mean amplitude. **(B)** Take lg (number of turns per second) as the independent variable and lg (mean amplitude) as the dependent variable for linear correlation analysis. A linear regression relationship exists between the two variables, with the linear regression equation of Y = a + b*X. **(C)** The standard error of intercept and slope of the linear regression equation are used to determine the upper and the lower boundaries of the cloud that contains the prediction interval of the mean amplitude corresponding to the number of turns per second. **(D)** After exponentiating the logarithm, the original data of the number of turns per second is used as the independent variable. The standard error of the intercept and slope are substituted into the linear regression equation. Each independent variable (the number of turns per second) has two corresponding dependent variables (the mean amplitude), as the upper and lower boundaries of the clouds. And the 99th percentile of the number of turns per second as the right boundaries of the clouds.

**Figure 2:**
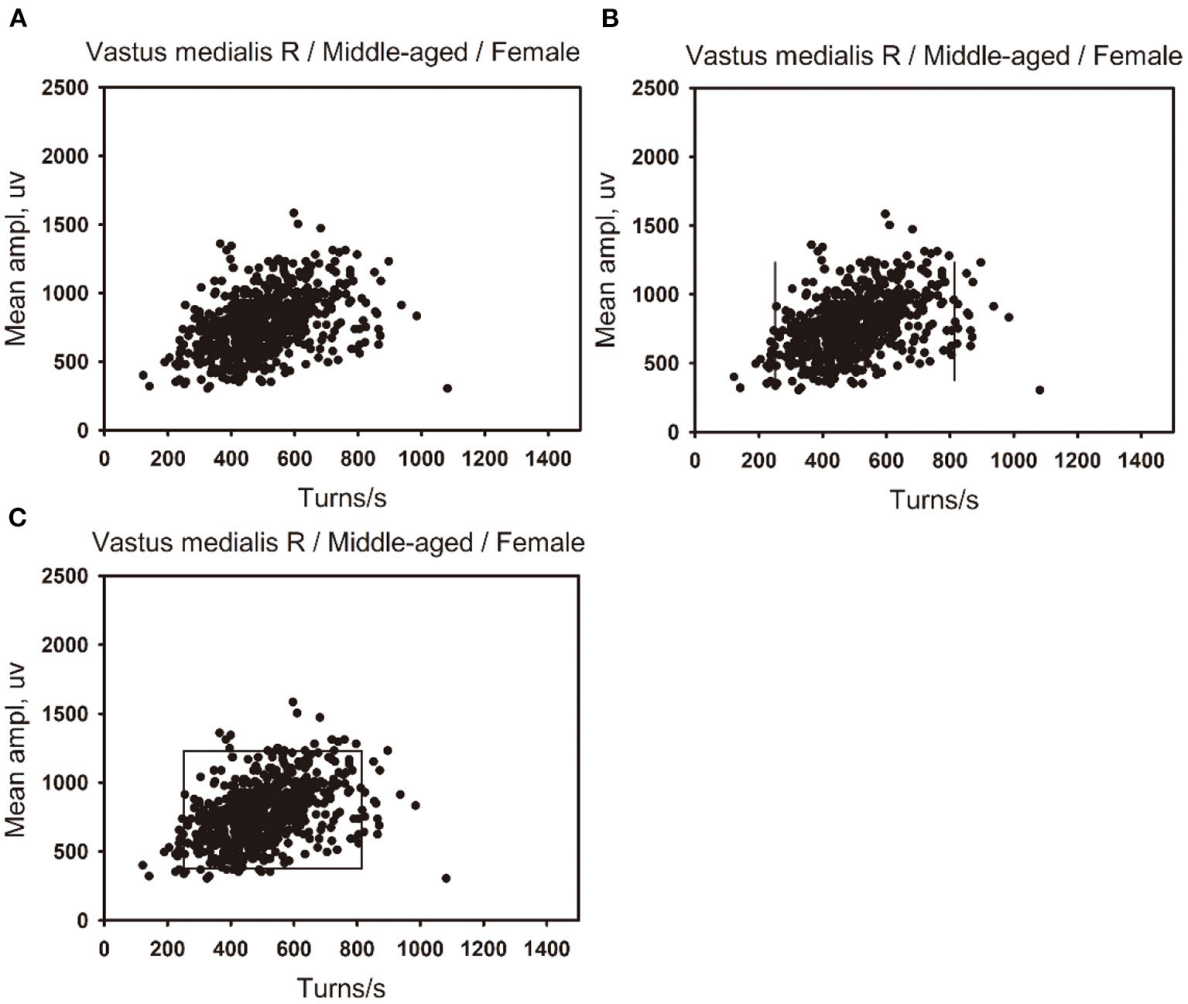
Developing method of the percentile clouds. **(A)** A scatter plot was made of the number of turns per second and the mean amplitude of 650 data points of the right vastus medialis in the middle-aged female group. **(B)** The 2.5 percentile of the number of turns per second is used as the left boundary of the clouds, and the 97.5 percentile is used as the right boundary of the clouds. **(C)** The 97.5 percentile of the mean amplitude is used as the upper boundary of the clouds, and the 2.5 percentile is used as the lower boundary of the clouds.

Two methods were exploited to develop clouds and establish reference values:

(1) The linear regression clouds: Referring to the turns-amplitude analysis technique created by Stålberg in 1983, with the number of turns per second as the independent variable and the mean amplitude as the dependent variable, a scatter plot was made (see Figure 1A), where it was found that for most muscles, the mean amplitude increased with the number of turns per second, and there was a positive linear correlation between the two variables. The normality tests were performed on the lg (number of turns per second) and lg (mean amplitude) of all muscles. As for the muscles with bivariate normal distribution and homogeneous variance, lg (number of turns per second) and lg (mean amplitude) were taken as the independent variable and the dependent variable, respectively, to make the scatter plot, which revealed that there was a linear positive correlation between the two variables.
Linear regression was performed on the lg (number of turns per second) and the lg (mean amplitude), the results of which showed that there was a linear regression relationship between the two variables in some muscles (see Figure 1B). X_0_ and Y_0_ denote the number of turns per second and mean amplitude for original data, respectively. X_0_ includes the minimum of the number of turns per second. By applying X = lg(X_0_) and Y = lg(Y_0_) to the linear regression equation, we obtained the relationship between X and Y for some muscles with the linear regression equation of Y=a+b^*^X. Consequently, the relationship corresponding to the original data is Y_0_ = 10^a^^*^X${}_{0}^{\text{b}}$. When the standard error (SE) of the intercept a and slope b are included in the relationship, we then obtained a new relationship: Y_0_ = [10^a(±SEa)^]^*^ (X_0_)^b(±SEb)^, where SEa and SEb were the standard error of the intercept and slope, respectively. It is obvious that there exist two Y_0_ values for each X_0_ when we calculated the prediction interval (Figure 1C). These two Y_0_ values correspond to the upper and lower boundaries of the clouds. Namely, we obtain two curves corresponding to two boundaries. With 99 percentiles of the number of turns per second as the right boundary, and the minimum value of the number of turns per second of the muscle as the left boundary, the cloud was obtained (see Figure 1D). The intercept, slope, standard error of the intercept and slope, and 99 percentiles of the number of turns per second were used as reference values for linear regression clouds.
(2) The percentile clouds: The percentile clouds can be applied, whereas the linear regression clouds cannot for all muscles including these three cases: (1) if lg (number of turns per second) and lg (mean amplitude) were not normally distributed; (2) there was no linear correlation between the two variables; (3) the two variables were linearly related, but the linear regression equation was not statistically significant. A scatter plot was drawn in Figure 2A. The 2.5 and 97.5 percentiles of the number of turns per second were used as the left and right boundaries of the clouds (see Figure 2B), and the 2.5 and 97.5 percentiles of the mean amplitude were used as the upper and lower boundaries of the clouds (see Figure 2C). A cloud was drawn to include 95% of the normal data records. The 2.5 and 97.5 percentiles of the number of turns per second and the mean amplitude were used as reference values of the percentile clouds.

### Determining the judgment criteria

For the muscles with a linear positive correlation between lg (number of turns per second) and lg (mean amplitude), the linear regression clouds were drawn. Meanwhile, the percentile clouds were drawn on all the muscle data, and the percentage of data records beyond the boundaries of the two clouds was calculated. The percentage was exploited to draw ROCs. Then, taking priority of specificity for determining criteria, specificity >94% was taken as a criterion to determine the optimum cut-off value according to ROCs, which can be used to discriminate between neuropathic lesion vs. non-neuropathic lesion, myopathic lesion vs. non-myopathic lesion, and abnormal muscles vs. normal muscles. According to the criteria, the sensitivity and specificity of the linear regression clouds, the percentile clouds, and QMUP were calculated. According to the area under the curve (AUC) of ROCs, the diagnostic values of the two methods were judged.

The clinical diagnosis was taken as the gold standard, and the sensitivity and specificity of the linear regression clouds, percentile clouds, and QMUP were investigated. Also, the AUC and Youden's index of the linear regression clouds and percentile clouds were compared.

### Statistical analysis

SPSS version 26.0 software (IBM Corp., Armonk, NY, USA) was used to determine whether lg (number of turns per second) and lg (mean amplitude) were normally distributed and whether the variances were homogeneous. According to the results, *t*-test, one-way analysis of variance or Wilcoxon rank sum test or Kruskal-Wallis *H* test were adopted to determine whether there are statistical differences in the lg (number of turns per second) and lg (mean amplitude) of different genders groups, different ages groups, and dominant limb muscles and non-dominant limb muscles groups, which with statistical differences of the results, were further analyzed through multiple linear regression. The linear correlation analysis was performed on lg (number of turns per second) and lg (mean amplitude). If there was a linear positive correlation between the two variables, the least square method was used for linear regression. Besides, hypothesis tests were performed on linear correlation coefficient, linear regression equation, and regression coefficient, with α = 0.05. When *P* ≤ 0.05, the results indicated that lg (number of turns per second) and lg (mean amplitude) had a linear regression relationship.

Then, a chi-square test with two degrees of freedom was performed to evaluate the significance of the sensitivity and specificity of the three methods of linear regression cloud, the percentile clouds, and QMUP. It was shown that when *P* ≤ 0.05, the difference was statistically significant.

## Results

### Subject demographic data

A total of 107 healthy subjects were recruited, and the data of electromyographic interference patterns were collected to establish reference values of clouds. According to gender and age, the subjects were grouped, as seen in Table 1. Meanwhile, a total of 249 inpatients were recruited. 190 normal muscles, 276 neuropathic lesion muscles, and 43 myopathic lesion muscles were collected. The demographic data were listed in Table 2, which is used to analyze the sensitivity and specificity of the linear regression clouds, percentile clouds, and QMUP.

**Table 1:** Demographic data of healthy subjects.

**Groups**	**No. of**	**Gender**	**Mean age**
**(years)**	**subjects**	**(Male/Female)**	**(years, mean ±SD)**
Young group (18–44)	32	15/17	35.40 ± 6.33
Middle-aged group (45–59)	48	17/31	47.82 ± 2.56
Older group (≥60)	27	15/12	62.47 ± 2.88
Total	107	47/60	

**Table 2:** Clinical diagnosis and the number of muscles sample.

**Diagnosis**	**Number of subjects**	**Age range**	**ADM**	**Biceps**	**EDC**	**Deltoid**	**Tibialis**	**Gastrocnemius**	**Vastus**	**SCM**	**Total number**
	**(male/female)**	**(years)**		**brachii**			**anterior**		**medialis**		**of muscles**
**Neuropathic**	142 (82/60)		91	17	6	20	56	40	41	5	276
Mononeuropathy	75 (43/32)	40–63	62	0	0	12	5	1	1	0	81
Polyneuropathy	16 (8/8)	18–60	21	0	0	0	21	16	4	0	62
Radiculopathy	46 (28/18)	32–70	4	7	6	6	25	22	27	0	97
ALS	5 (3/2)	45–52	4	10	0	2	5	1	9	5	36
**Myopathic**	15 (4/11)		0	5	3	12	8	4	11	0	43
Dermatomyositis	3 (0/3)	20–30	0	1	0	4	0	0	3	0	8
Polymyositis	11 (3/8)	18–60	0	3	3	7	6	3	7	0	29
Progressive muscular dystrophy	1 (1/0)	19	0	1	0	1	2	1	1	0	6
**Normal**	92 (47/45)		17	7	24	20	40	27	45	10	190
Healthy	49 (29/20)	23–63	13	5	14	11	28	16	27	7	121
Low calcium seizures	2 (2/0)	40–49	0	0	0	2	2	2	0	0	6
Anxiety	41 (16/25)	37–61	4	2	10	7	10	9	18	3	63
Total	249 (133/116)	18–63	108	29	33	52	104	71	97	15	509

ADM, abductor digiti minimi; ALS, amyotrophic lateral sclerosis; EDC, extensor digitorum communis; SCM, sternocleidomastoid.

### The reference values for two clouds were established

**Figure 3:**
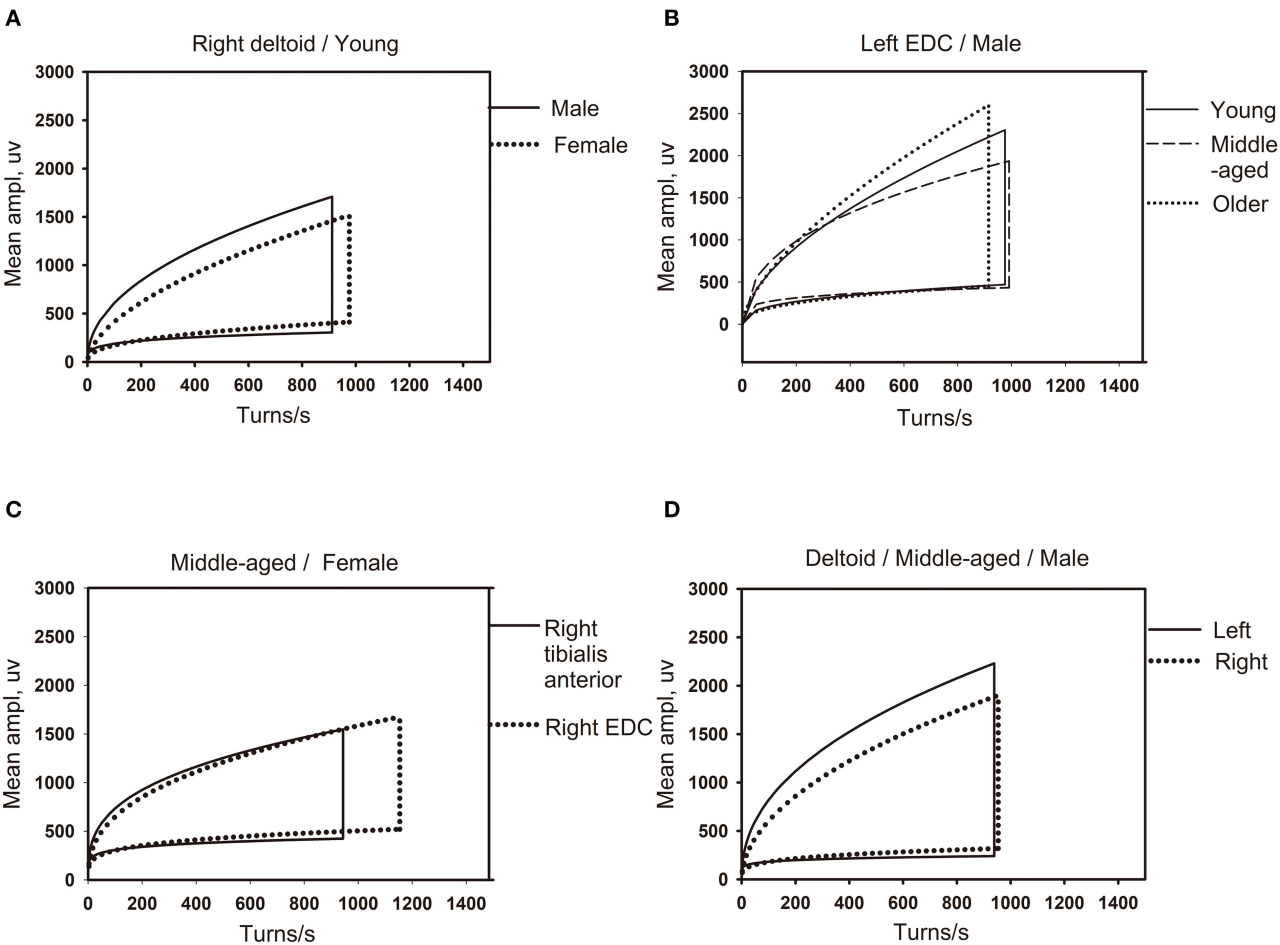
The influence of different factors on the shape of the linear regression clouds. **(A)** The clouds of different gender for same muscle (right deltoid) in the youth group (18–44 years old). **(B)** The clouds of different age groups for the same gender (male) in the same muscle of left extensor digitorum communis (EDC). **(C)** The clouds of different muscles (right tibialis anterior and right EDC) for the same gender (female) in middle-aged group (45–49 years old). **(D)** The clouds of left and right limb muscles (left and right deltoid) for the same gender (male) in middle-aged group (45–49 years old). EDC, extensor digitorum communis.

**Figure 4:**
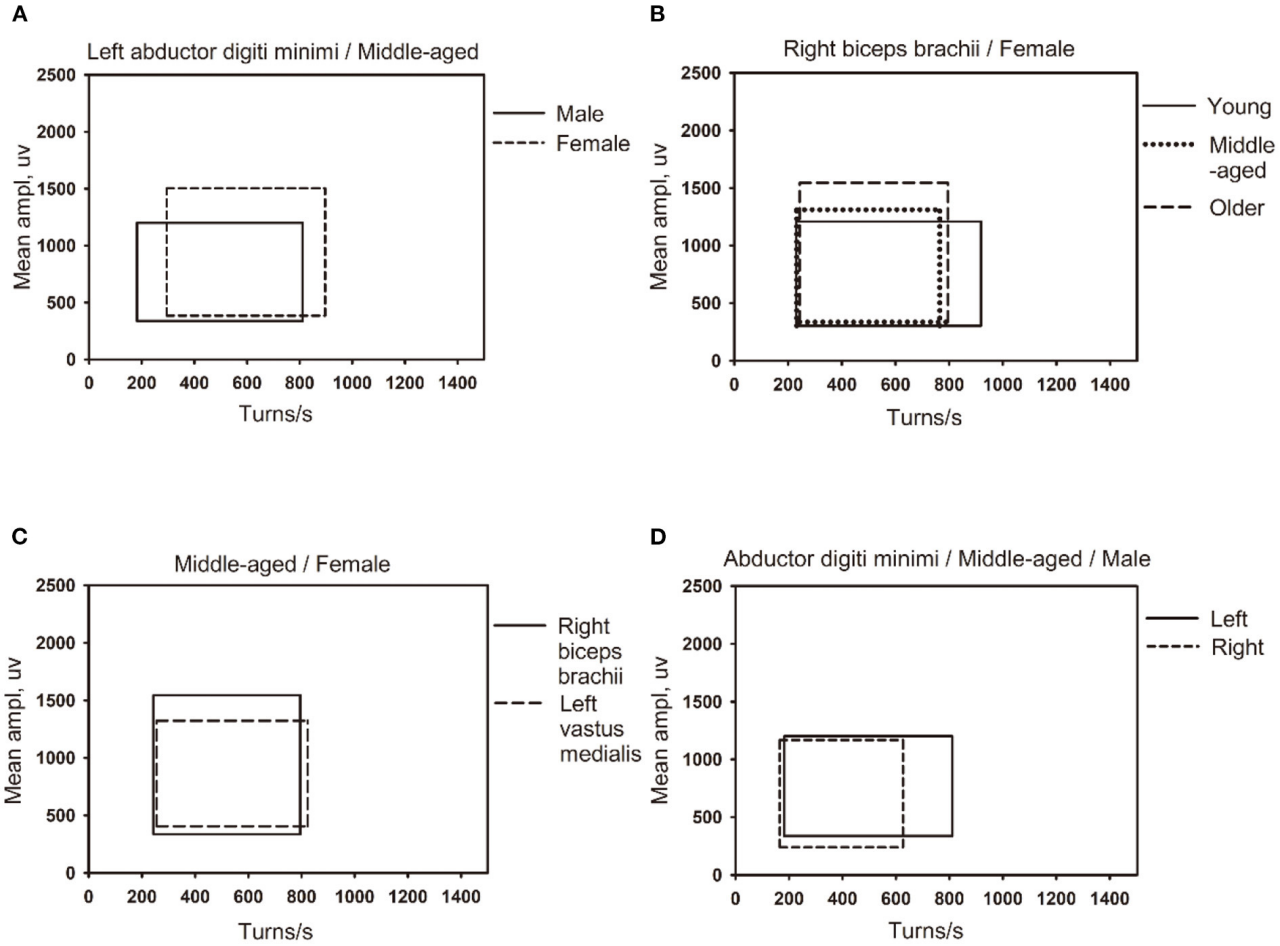
The influence of different factors on the shape of the percentile clouds. **(A)** The clouds of different gender for same muscle (left abductor digiti minimi) in the middle-aged group (45–49 years old). **(B)** The clouds of different age groups for the same gender (female) in the same muscle (right biceps brachii muscle). **(C)** The clouds of different muscles (right biceps brachii and left vastus medialis) for the same gender (female) in the middle-aged group (45–49 years old). **(D)** The clouds of left and right muscles (left and right abductor digiti minimi) for the same gender (male) in middle-aged group (45–49 years old).

The lg (number of turns per second) and lg (mean amplitude) of different genders, ages, and the dominant limb muscles and non-dominant limb muscles were compared, and the differences were statistically significant (see Tables 3–6). Besides, there were significant differences between the linear regression clouds and percentile clouds (see Figures 3, 4) of different genders, ages, and the dominant limb muscles and non-dominant limb muscles. The reference values for the linear regression clouds and percentile clouds were established for different groups (see Tables 7, 8).

**Table 3:** The comparison of the results of lg (number of turns per second) and lg (mean amplitude) for different genders groups.

**Muscle**	**Age**	**Data point**	**Data point**	**lg (number of**			**lg**		
	**(years)**	**of male**	**of female**	**turns per second)**			**(mean amplitude)**		
				***t*/*t^′^***	**Z**	**P**	***t*/*t^′^***	**Z**	**P**
Non-dominant ADM	18–44	279	317		−1.673	0.094	1.816		0.070
	45–59	338	620		−7.826	<0.001^#^		−8.571	<0.001^#^
	≥60	296	240	4.736*		<0.001^#^	7.146		<0.001^#^
Dominant ADM	18–44	274	331		−4.025	<0.001^#^	−1.026		0.305
	45–59	318	618	−13.216		<0.001^#^		−11.136	<0.001^#^
	≥60	256	239	−3.696		<0.001^#^	3.196		0.001^#^
Non-dominant EDC	18–44	300	338		−4.205	<0.001^#^	9.611*		<0.001^#^
	45–59	340	617	−6.034*		<0.001^#^	0.021*		0.983
	≥60	298	238		−3.941	<0.001^#^	6.596*		<0.001^#^
Dominant EDC	18–44	298	340		−4.808	<0.001^#^	7.307		<0.001^#^
	45–59	320	614		−10.079	<0.001^#^	−9.105		<0.001^#^
	≥60	283	238	−2.666*		0.008^#^	8.989		<0.001^#^
Non-dominant biceps brachii	18–44	297	339	1.604*		0.109		−8.926	<0.001^#^
	45–59	340	613	−3.660*		<0.001^#^		−0.870	0.384
	≥60	298	234	4.009*		<0.001^#^		−4.907	<0.001^#^
Dominant biceps brachii	18–44	300	338		−4.319	<0.001^#^		−9.392	<0.001^#^
	45–59	338	615		−3.770	<0.001^#^	3.538*		<0.001^#^
	≥60	295	239	−1.660		0.097		−3.495	<0.001^#^
Non-dominant deltoid	18–44	300	339	4.506		<0.001^#^		−2.572	0.010^#^
	45–59	340	616	1.748*		0.081	−7.570*		<0.001^#^
	≥60	294	234		−1.885	0.059	3.324*		0.001^#^
Dominant deltoid	18–44	298	329	7.115*		<0.001^#^	3.549*		<0.001^#^
	45–59	319	606	−2.11		0.027^#^	−5.658*		<0.001^#^
	≥60	297	239		−3.313	0.001^#^	−2.690*		0.007^#^
Non-dominant tibialis anterior	18–44	300	338		−2.199	0.028^#^	10.630*		<0.001^#^
	45–59	340	616	−5.237*		<0.001^#^		−6.715	<0.001^#^
	≥60	259	239		−2.081	0.037^#^	3.732		<0.001^#^
Dominant tibialis anterior	18–44	299	338	−0.316		0.752	7.489		<0.001^#^
	45–59	340	615	−4.793*		<0.001^#^		−2.321	0.020^#^
	≥60	258	239	−3.374		0.001^#^	5.090		<0.001^#^
Non-dominant gastrocnemius	18–44	277	337		−2.442	0.015^#^	2.961		0.003^#^
	45–59	339	619		−6.656	<0.001^#^		−4.438	<0.001^#^
	≥60	280	240		−2.761	0.006^#^	1.581*		0.114
Dominant gastrocnemius	18–44	280	335		−0.969	0.333		−0.077	0.939
	45–59	319	611		−4.926	<0.001^#^		−2.633	0.008^#^
	≥60	260	240	−2.591		0.010^#^	4.314		<0.001^#^
Non-dominant vastus medialis	18–44	279	338	5.477*		<0.001^#^		−6.403	<0.001^#^
	45–59	339	612		−5.388	<0.001^#^	−6.742*		<0.001^#^
	≥60	240	238		−0.869	0.385	5.174		<0.001^#^
Dominant vastus medialis	18–44	277	340		−5.270	<0.001^#^	7.431		<0.001^#^
	45–59	340	615	−5.875*		<0.001^#^		−2.691	0.007^#^
	≥60	277	240	−3.843*		<0.001^#^	0.035		0.972
Non-dominant SCM	18–44	298	320	1.492		0.136		−4.290	<0.001^#^
	45–59	339	618		−6.770	<0.001^#^		−1.859	0.063
	≥60	299	220	1.625		0.105	8.820		<0.001^#^
Dominant SCM	18–44	299	339		−3.311	0.001^#^		−5.745	<0.001^#^
	45–59	337	620		−7.625	<0.001^#^		−7.121	<0.001^#^
	≥60	300	220	2.620		0.009^#^		−6.082	<0.001^#^

ADM, abductor digiti minimi; EDC, extensor digitorum communis; SCM, sternocleidomastoid.

^*^using t-test,

^#^p ≤ 0.05.

**Table 4:** The comparison of the results of lg (number of turns per second) and lg (mean amplitude) for different age groups.

**Muscle**	**Gender**	**Data point for**	**Data point for the**	**Data point for**	**lg (number of turns**		**lg**	
		**the youth group**	**middle-aged group**	**the older group**	**per second)**		**(mean amplitude)**	
					***F*/*H***	**P**	***F*/*H***	**P**
Non-dominant ADM	Male	279	338	296	13.452	0.001^#^	11.694	0.003
	Female	317	620	240	134.931	<0.001^#^	150.096	<0.001^#^
Dominant ADM	Male	274	318	256	22.276	<0.001^#^	9.623	0.008^#^
	Female	331	618	239	131.440	<0.001^#^	206.601	<0.001^#^
Non-dominant EDC	Male	300	340	298	20.136	<0.001^#^	0.068	0.967
	Female	338	617	238	133.337	<0.001^#^	103.688	<0.001^#^
Dominant EDC	Male	298	320	283	1.040	0.595	41.544	<0.001^#^
	Female	340	614	238	201.605*	<0.001^#^	202.141	<0.001^#^
Non-dominant biceps brachii	Male	297	340	298	0.761	0.683	24.766	<0.001^#^
	Female	339	613	234	91.274	<0.001^#^	85.833	<0.001^#^
Dominant biceps brachii	Male	300	338	295	21.322	<0.001^#^	61.641	<0.001^#^
	Female	338	615	239	29.347	<0.001^#^	14.206	0.001^#^
Non-dominant deltoid	Male	300	340	294	38.493	<0.001^#^	25.860	<0.001^#^
	Female	339	616	234	1.425	0.241	61.923	<0.001^#^
Dominant deltoid	Male	298	319	297	9.738	0.008^#^	3.647	0.159
	Female	329	606	239	63.710	<0.001^#^	50.913*	<0.001^#^
Non-dominant tibialis anterior	Male	300	340	259	0.126	0.939	39.262	<0.001^#^
	Female	338	616	239	8.231	0.016^#^	161.899	<0.001^#^
Dominant tibialis anterior	Male	299	340	258	4.482	0.106	5.034	0.081
	Female	338	615	239	2.562	0.078	24.212*	<0.001^#^
Non-dominant gastrocnemius	Male	277	339	280	2.865	0.239	3.981*	0.019^#^
	Female	337	619	240	139.895	<0.001^#^	150.280	<0.001^#^
Dominant gastrocnemius	Male	280	319	260	15.091	0.001^#^	50.960	<0.001^#^
	Female	335	611	240	83.654	<0.001^#^	83.519	<0.001^#^
Non-dominant vastus medialis	Male	279	339	240	3.315	0.191	10.599	0.005^#^
	Female	338	612	238	83.993	<0.001^#^	161.100	<0.001^#^
Dominant vastus medialis	Male	277	340	277	19.772	<0.001^#^	11.855*	<0.001^#^
	Female	340	615	240	204.783	<0.001^#^	157.116	<0.001^#^
Non-dominant SCM	Male	298	339	299	4.921	0.085	11.477	0.003^#^
	Female	320	618	220	194.646	<0.001^#^	164.849	<0.001^#^
Dominant SCM	Male	299	337	300	8.547	0.014^#^	32.634	<0.001^#^
	Female	339	620	220	111.374	<0.001^#^	99.114	<0.001^#^

ADM, abductor digiti minimi; EDC, extensor digitorum communis; SCM, sternocleidomastoid.

^*^Using one-way analysis of variance,

^#^P ≤ 0.05.

**Table 5:** The comparison of the results of lg (number of turns per second) and lg (mean amplitude) for the non-dominant and dominant muscles.

**Muscle**	**Gender**	**Age**	**Non-dominant**	**Dominant**	**lg (number of**			**lg**		
		**(years)**	**data point**	**data point**	**turns per second)**			**(mean amplitude)**		
					***t*/*t^′^***	**Z**	**P**	***t*/*t^′^***	**Z**	**P**
ADM	Male	18–44	279	274	9.307*		<0.001^#^	5.968		<0.001^#^
		45–59	338	318		−5.178	<0.001^#^		−4.917	<0.001^#^
		≥60	296	256	7.445*		<0.001^#^	6.650		<0.001^#^
	Female	18–44	317	331		−3.131	0.002^#^	3.843		<0.001^#^
		45–59	620	618	3.080		0.002^#^		−1.554	0.120
		≥60	240	239	−1.022		0.307	2.365		0.018^#^
EDC	Male	18–44	300	298	−0.633		0.527	6.966		<0.001^#^
		45–59	340	320		−1.137	0.256	6.555		<0.001^#^
		≥60	298	283	−3.964		<0.001^#^	0.923*		0.356
	Female	18–44	338	340		−0.682	0.495	3.536*		<0.001^#^
		45–59	617	614	−2.896*		0.004^#^	−1.815*		0.070
		≥60	238	238		−2.163	0.031^#^	2.209		0.028^#^
Biceps brachii	Male	18–44	297	300	−3.819*		<0.001^#^	−2.745		0.006^#^
		45–59	340	338	0.718		0.473		−0.021	0.983
		≥60	298	295	1.239		0.216	0.767		0.443
	Female	18–44	339	338		−2.315	0.019^#^		−2.408	0.016^#^
		45–59	613	615		−0.862	0.388	4.436		<0.001^#^
		≥60	234	239	−4.346*		<0.001^#^		−0.498	0.618
Deltoid	Male	18–44	300	298	3.250		0.001^#^		−7.793	<0.001^#^
		45–59	340	319	4.252		<0.001^#^	1.842		0.066
		≥60	294	297		−1.231	0.218	7.420*		<0.001^#^
	Female	18–44	339	329	6.155*		<0.001^#^	8.401		<0.001^#^
		45–59	616	606	0.937*		0.349	4.763*		<0.001^#^
		≥60	234	239	0.052		0.958	0.050		0.960
Tibialis anterior	Male	18–44	300	299		−0.405	0.685	−0.176		0.860
		45–59	340	340	1.614		0.107		−5.065	<0.001^#^
		≥60	259	258	0.867*		0.386	1.796		0.073
	Female	18–44	338	338		−1.328	0.184	−3.441*		0.001^#^
		45–59	616	615	3.041		0.002^#^	3.285		0.001^#^
		≥60	239	239		−0.279	0.780	3.450*		0.001^#^
Gastrocnemius	Male	18–44	277	280		−1.123	0.261		−0.417	0.677
		45–59	339	319	0.495		0.621		−1.656	0.098
		≥60	280	260	−1.724		0.085	−3.885		<0.001^#^
	Female	18–44	337	335		−0.515	0.606		−2.720	0.007^#^
		45–59	619	611		−2.228	0.026^#^		−0.364	0.716
		≥60	240	240		−1.986	0.047^#^	−1.133*		0.258
Vastus medialis	Male	18–44	279	277	5.355		<0.001^#^		−1.372	0.170
		45–59	339	340	1.212		0.226	−2.966*		0.003^#^
		≥60	240	277		−5.365	<0.001^#^	3.515		<0.001^#^
	Female	18–44	338	340		−4.897	<0.001^#^	2.128		0.034^#^
		45–59	612	615		−2.155	0.031^#^		−1.622	0.105
		≥60	238	240	2.743		0.006^#^	−1.662		0.097
SCM	Male	18–44	298	299	−9.473		<0.001^#^		−5.785	<0.001^#^
		45–59	339	337		−1.082	0.279		−2.487	0.013^#^
		≥60	299	300	−4.437		<0.001^#^	−1.782		0.075
	Female	18–44	320	339		−5.808	<0.001^#^		−3.092	0.002^#^
		45–59	618	620	−4.011		<0.001^#^	−3.370		0.001^#^
		≥60	220	220	−3.024		0.003^#^		−4.121	<0.001^#^

ADM, abductor digiti minimi; EDC, extensor digitorum communis; SCM, sternocleidomastoid.

^*^Using t-test,

^#^P ≤ 0.05.

**Table 6:** The comparison of the results of lg (number of turns per second) and lg (mean amplitude) using multiple linear regression model.

		**lg(number of turns per second)**				**lg(mean amplitude)**			
**Muscle**	**Factors**	**R^2^**	**B**	***t***	***p***	**R^2^**	**B**	***t***	***p***
ADM	Gender	0.118	−0.010	−1.115	0.265	0.129	−0.060	−6.311	<0.001^#^
	Dominant vs. non-dominant	0.118	−0.045	−10.394	<0.001^#^	0.129	−0.046	−9.702	<0.001^#^
	Young man vs. old man	0.118	−0.043	−5.091	<0.001^#^	0.129	−0.036	−3.895	<0.001^#^
	Middle-Aged man vs. old man	0.118	−0.028	−3.451	0.001^#^	0.129	−0.032	−3.583	<0.001^#^
	Young man vs. middle-aged man	0.118	0.015	1.854	0.064	0.129	0.004	0.475	0.635
	Young woman vs. old woman	0.118	−0.023	−2.773	0.006^#^	0.129	0.019	2.082	0.037^#^
	Middle-Aged woman vs. old woman	0.118	0.084	11.060	<0.001^#^	0.129	0.140	17.123	<0.001^#^
	Young woman vs. middle-aged woman	0.118	0.107	15.720	<0.001^#^	0.129	0.121	16.415	<0.001^#^
EDC	Gender	0.107	0.033	3.664	<0.001^#^	0.118	−0.092	−10.045	<0.001^#^
	Dominant vs. non-dominant	0.107	0.012	2.621	0.009^#^	0.118	−0.029	−6.137	<0.001^#^
	Young man vs. old man	0.107	0.030	3.516	<0.001^#^	0.118	−0.029	−3.319	0.001^#^
	Middle-Aged man vs. old man	0.107	0.033	3.973	<0.001^#^	0.118	−0.026	−3.119	0.002^#^
	Young man vs. middle-aged man	0.107	0.003	0.376	0.707	0.118	0.002	0.282	0.778
	Young woman vs. old woman	0.107	−0.058	−6.728	<0.001^#^	0.118	−0.040	−4.442	<0.001^#^
	Middle-Aged woman vs. old woman	0.107	0.080	10.314	<0.001^#^	0.118	0.106	13.264	<0.001^#^
	Young woman vs. middle-aged woman	0.107	0.138	20.045	<0.001^#^	0.118	0.146	20.515	<0.001^#^
Biceps brachii	Gender	0.023	−0.018	−1.982	0.048^#^	0.060	−0.053	−5.373	<0.001^#^
	Dominant vs. non-dominant	0.023	0.012	2.623	0.009^#^	0.060	−0.005	−1.073	0.283
	Young man vs. old man	0.023	0.021	2.550	0.011^#^	0.060	0.087	9.356	<0.001^#^
	Middle-Aged man vs. old man	0.023	0.010	1.227	0.220	0.060	0.035	3.868	<0.001^#^
	Young man vs. middle-aged man	0.023	−0.011	−1.402	0.161	0.060	−0.052	−5.781	<0.001^#^
	Young woman vs. old woman	0.023	0.008	0.892	0.373	0.060	0.021	2.203	0.028^#^
	Middle-Aged woman vs. old woman	0.023	0.061	7.830	<0.001^#^	0.060	0.062	7.048	<0.001^#^
	Young woman vs. middle-aged woman	0.023	0.053	7.736	<0.001^#^	0.060	0.040	5.209	<0.001^#^
Deltoid	Gender	0.033	0.024	3.668	<0.001^#^	0.071	−0.006	−0.682	0.495
	Dominant vs. non-dominant	0.033	−0.022	−6.736	<0.001^#^	0.071	−0.049	−11.816	<0.001^#^
	Young man vs. old man	0.033	0.037	5.864	<0.001^#^	0.071	0.011	1.479	0.139
	Middle-Aged man vs. old man	0.033	0.020	3.264	0.001^#^	0.071	−0.007	−0.921	0.357
	Young man vs. middle-aged man	0.033	−0.017	−2.748	0.006^#^	0.071	−0.018	−2.443	0.015^#^
	Young woman vs. old woman	0.033	−0.041	−6.375	<0.001^#^	0.071	−0.012	−1.551	0.121
	Middle-Aged woman vs. old woman	0.033	−0.003	−0.500	0.617	0.071	0.061	8.395	<0.001^#^
	Young woman vs. middle-aged woman	0.033	0.038	7.398	<0.001^#^	0.071	0.073	11.385	<0.001^#^
Tibialis anterior	Gender	0.019	0.027	3.420	0.001^#^	0.053	−0.049	−5.648	<0.001^#^
	Dominant vs. non-dominant	0.019	−0.010	−2.520	0.012^#^	0.053	0.001	0.151	0.880
	Young man vs. old man	0.019	0.007	0.979	0.328	0.053	0.016	1.904	0.057
	Middle-Aged man vs. old man	0.019	−0.005	−0.683	0.495	0.053	−0.022	−2.733	0.006^#^
	Young man vs. middle-aged man	0.019	−0.012	−1.760	0.079	0.053	−0.037	−4.885	<0.001^#^
	Young woman vs. old woman	0.019	−0.009	−1.251	0.211	0.053	−0.032	−4.001	<0.001^#^
	Middle-Aged woman vs. old woman	0.019	0.013	2.006	0.045^#^	0.053	0.050	6.835	<0.001^#^
	Young woman vs. middle-aged woman	0.019	0.023	3.821	<0.001^#^	0.053	0.082	12.690	<0.001^#^
Gastrocnemius	Gender	0.067	0.031	3.400	0.001^#^	0.066	−0.040	−4.107	<0.001^#^
	Dominant vs. non-dominant	0.067	−0.002	−0.367	0.714	0.066	0.015	2.986	0.003^#^
	Young man vs. old man	0.067	−0.034	−3.884	<0.001^#^	0.066	−0.068	−7.234	<0.001^#^
	Middle-Aged man vs. old man	0.067	0.001	0.113	0.910	0.066	−0.014	−1.515	0.130
	Young man vs. middle-aged man	0.067	0.035	4.188	<0.001^#^	0.066	0.055	6.060	<0.001^#^
	Young woman vs. old woman	0.067	−0.086	−9.815	<0.001^#^	0.066	−0.043	−4.652	<0.001^#^
	Middle-Aged woman vs. old woman	0.067	0.022	2.802	0.005^#^	0.066	0.066	7.890	<0.001^#^
	Young woman vs. middle-aged woman	0.067	0.108	15.372	<0.001^#^	0.066	0.110	14.648	<0.001^#^
Vastus medialis	Gender	0.091	0.035	3.550	<0.001^#^	0.076	−0.031	−3.462	0.001^#^
	Dominant vs. non-dominant	0.091	−0.040	−8.413	<0.001^#^	0.076	−0.009	−2.092	0.036^#^
	Young man vs. old man	0.091	0.039	4.175	<0.001^#^	0.076	0.036	4.286	<0.001^#^
	Middle-Aged man vs. old man	0.091	0.041	4.581	<0.001^#^	0.076	0.014	1.727	0.084
	Young man vs. middle-aged man	0.091	0.002	0.215	0.830	0.076	−0.022	−2.817	0.005^#^
	Young woman vs. old woman	0.091	−0.069	−7.499	<0.001^#^	0.076	−0.021	−2.527	0.012^#^
	Middle-Aged woman vs. old woman	0.091	0.060	7.252	<0.001^#^	0.076	0.089	11.840	<0.001^#^
	Young woman vs. middle-aged woman	0.091	0.128	17.530	<0.001^#^	0.076	0.110	16.494	<0.001^#^
SCM	Gender	0.095	−0.027	−2.832	0.005^#^	0.076	−0.082	−9.541	<0.001^#^
	Dominant vs. non-dominant	0.095	0.044	9.347	<0.001^#^	0.076	0.020	4.745	<0.001^#^
	Young man vs. old man	0.095	0.003	0.395	0.693	0.076	−0.012	−1.560	0.119
	Middle-Aged man vs. old man	0.095	−0.026	−3.073	0.002^#^	0.076	−0.034	−4.441	<0.001^#^
	Young man vs. middle-aged man	0.095	−0.029	−3.476	0.001^#^	0.076	−0.022	−2.831	0.005^#^
	Young woman vs. old woman	0.095	−0.004	−0.458	0.647	0.076	0.009	1.082	0.279
	Middle-Aged woman vs. old woman	0.095	0.104	12.398	<0.001^#^	0.076	0.104	13.770	<0.001^#^
	young woman vs. Middle-aged woman	0.095	0.108	14.854	<0.001^#^	0.076	0.095	14.467	<0.001^#^

ADM, abductor digiti minimi; EDC, extensor digitorum communis; SCM, sternocleidomastoid.

^#^P ≤ 0.05.

**Table 7:** The reference values of the linear regression clouds.

**Muscle**	**Gender**	**Age (years)**	**Data record (number)**	**Intercept**	**Standard error of intercept**	**Slope**	**Standard error of slope**	**99 percentiles of the number of turns per second**	**Maximum value of the number of turns per second**	**Maximum value of the mean amplitude**	***R^2^***
Non-dominant ADM	Male	18–44	279	1.461	0.222	0.513	0.084	806	827	1,456	0.119
		45–59									
		≥60									
	Female	18–44									
		45–59									
		≥60									
Dominant ADM	Male	18–44	274	1.201	0.150	0.603	0.059	636	705	1,680	0.277
		45–59									
		≥60	256	1.891	0.147	0.340	0.057	686	765	1,072	0.124
	Female	18–44									
		45–59	618	1.660	0.132	0.453	0.049	944	1,077	1,856	0.123
		≥60	239	2.213	0.162	0.199	0.062	729	752	1,104	0.042
Non-dominant EDC	Male	18–44	300	1.620	0.159	0.467	0.059	976	1,045	1,632	0.175
		45–59	340	2.023	0.150	0.314	0.055	991	1,125	1,600	0.087
		≥60	298	1.499	0.172	0.519	0.065	915	960	1,712	0.178
	Female	18–44									
		45–59	617	1.280	0.110	0.575	0.040	1,198	1,345	1,952	0.256
		≥60									
Dominant EDC	Male	18–44	298	1.495	0.137	0.482	0.051	1,023	1,167	1,456	0.235
		45–59									
		≥60	283	2.020	0.171	0.313	0.063	788	792	1,536	0.080
	Female	18–44									
		45–59	614	2.039	0.118	0.304	0.042	1,154	1,355	1,840	0.078
		≥60	238	1.785	0.117	0.359	0.043	1,273	1,387	1,056	0.230
Non-dominant biceps brachii	Male	18–44	297	1.798	0.135	0.417	0.051	1,021	1,055	1,824	0.186
		45–59									
		≥60	298	1.305	0.139	0.577	0.052	858	1,025	1,840	0.292
	Female	18–44									
		45–59	613	2.304	0.128	0.206	0.047	857	967	1,760	0.030
		≥60									
Dominant biceps brachii	Male	18–44	300	1.612	0.151	0.492	0.056	985	1,092	1,872	0.205
		45–59	338	1.747	0.168	0.422	0.063	1,016	1,117	1,968	0.117
		≥60	295	1.202	0.150	0.615	0.057	825	830	1,840	0.286
	Female	18–44									
		45–59									
		≥60									
Non-dominant deltoid	Male	18–44									
		45–59	340	2.032	0.227	0.284	0.083	939	1,000	1,616	0.034
		≥60									
	Female	18–44									
		45–59	616	2.540	0.127	0.126	0.047	1,033	1,265	1,632	0.012
		≥60	234	2.339	0.235	0.170	0.086	982	1,067	1,792	0.017
Dominant deltoid	Male	18–44	298	1.854	0.176	0.340	0.064	912	1,025	1,312	0.087
		45–59	319	1.769	0.179	0.377	0.066	954	1,065	1,408	0.094
		≥60									
	Female	18–44	329	1.484	0.129	0.474	0.048	971	1,040	1,376	0.228
		45–59									
		≥60	239	1.761	0.230	0.381	0.084	911	962	1,408	0.080
Non-dominant tibialis anterior	Male	18–44									
		45–59									
		≥60	259	1.370	0.128	0.558	0.048	851	865	1,536	0.346
	Female	18–44									
		45–59	616	2.023	0.130	0.311	0.048	909	1,007	1,664	0.065
		≥60									
Dominant tibialis anterior	Male	18–44	299	1.568	0.167	0.486	0.062	845	907	1,600	0.171
		45–59									
		≥60	258	1.379	0.172	0.549	0.065	860	922	1,488	0.221
	Female	18–44	338	2.180	0.157	0.228	0.058	906	985	1,360	0.044
		45–59	615	2.202	0.130	0.237	0.048	944	1,055	1,568	0.038
		≥60	239	1.786	0.186	0.370	0.069	926	1,052	1,264	0.109
Non-dominant gastrocnemius	Male	18–44									
		45–59	339	1.292	0.139	0.576	0.052	950	1,057	1,616	0.269
		≥60	280	0.868	0.159	0.737	0.059	812	930	1,552	0.357
	Female	18–44	337	1.200	0.111	0.595	0.042	883	1,035	1,216	0.374
		45–59									
		≥60									
Dominant gastrocnemius	Male	18–44									
		45–59									
		≥60	260	0.689	0.147	0.818	0.054	849	860	1,920	0.467
	Female	18–44									
		45–59									
		≥60	240	1.559	0.163	0.466	0.060	998	1,117	1,424	0.203
Non-dominant vastus medialis	Male	18–44									
		45–59	339	1.413	0.128	0.532	0.048	890	920	1,664	0.266
		≥60									
	Female	18–44	338	1.494	0.087	0.496	0.034	896	1,055	1,408	0.394
		45–59									
		≥60	238	1.752	0.145	0.389	0.055	960	1,027	1,184	0.177
Dominant vastus medialis	Male	18–44	277	1.750	0.130	0.422	0.050	839	900	1,744	0.206
		45–59	340	1.197	0.099	0.630	0.038	893	932	1,504	0.454
		≥60	277	1.498	0.086	0.510	0.034	694	802	1,312	0.454
	Female	18–44									
		45–59									
		≥60	240	1.825	0.144	0.374	0.055	807	852	1,440	0.163
Non-dominant SCM	Male	18–44	298	1.546	0.138	0.421	0.052	831	977	976	0.184
		45–59									
		≥60	299	1.664	0.116	0.385	0.043	1,032	1,132	1,040	0.212
	Female	18–44									
		45–59									
		≥60	220	2.084	0.128	0.197	0.048	1,007	1,110	736	0.071
Dominant SCM	Male	18–44									
		45–59									
		≥60	300	1.240	0.122	0.540	0.044	997	1,025	1,120	0.332
	Female	18–44									
		45–	620	1.232	0.101	0.539	0.036	1,287	1,427	1,280	0.266
		≥60									

ADM, abductor digiti minimi; EDC, extensor digitorum communis; SCM, sternocleidomastoid.

**Table 8:** The reference values of the percentile clouds.

**Muscle**	**Gender**	**Age (years)**	**Data record (number)**	**2.5 percentiles of the number of turns per second**	**97.5 percentiles of the number of turns per second**	**2.5 percentiles of the mean amplitude**	**97.5 percentiles of the mean amplitude**
Non-dominant ADM	Male	18–44	279	250	727	288	1,280
		45–59	338	182	811	336	1,201
		≥60	296	226	846	375	1,401
	Female	18–44	317	174	748	335	1,216
		45–59	620	295	897	384	1,504
		≥60	240	210	735	288	1,024
Dominant ADM	Male	18–44	274	160	627	240	1,150
		45–59	318	165	627	240	1,168
		≥60	256	178	621	336	992
	Female	18–44	331	170	727	256	1,163
		45–59	618	286	877	360	1,512
		≥60	239	220	670	304	912
Non-dominant EDC	Male	18–44	300	230	836	360	1,424
		45–59	340	277	918	360	1,296
		≥60	298	204	782	343	1,464
	Female	18–44	338	243	790	272	1,192
		45–59	617	277	1,124	336	1,577
		≥60	238	137	965	304	1,056
Dominant EDC	Male	18–44	298	246	935	336	1,170
		45–59	320	225	797	352	1,104
		≥60	283	256	775	354	1,262
	Female	18–44	340	178	878	240	976
		45–59	614	350	1,094	400	1,424
		≥60	238	260	1,103	352	960
Non-dominant biceps brachii	Male	18–44	297	196	875	375	1,664
		45–59	340	245	971	376	1,560
		≥60	298	232	775	368	1,428
	Female	18–44	339	228	785	320	1,353
		45–59	613	283	825	368	1,477
		≥60	234	176	726	282	1,504
Dominant biceps brachii	Male	18–44	300	243	894	400	1,688
		45–59	338	213	931	296	1,728
		≥60	295	217	729	342	1,466
	Female	18–44	338	230	919	304	1,209
		45–59	615	243	795	336	1,546
		≥60	239	232	765	304	1,312
Non-dominant deltoid	Male	18–44	300	334	872	368	1,176
		45–59	340	356	863	352	1,280
		≥60	294	316	820	416	1,326
	Female	18–44	339	295	810	384	1,216
		45–59	616	240	895	448	1,337
		≥60	234	313	900	336	1,410
Dominant deltoid	Male	18–44	298	353	860	352	1,040
		45–59	319	332	892	368	1,104
		≥60	297	286	779	375	992
	Female	18–44	329	243	856	292	1,020
		45–59	606	340	825	352	1,408
		≥60	239	327	827	368	1,120
Non-dominant tibialis anterior	Male	18–44	300	258	792	368	1,456
		45–59	340	240	806	336	1,280
		≥60	259	248	805	448	1,264
	Female	18–44	338	238	859	336	1,016
		45–59	616	310	859	384	1,280
		≥60	239	237	825	400	1,104
Dominant tibialis anterior	Male	18–44	299	245	797	408	1,384
		45–59	340	221	852	360	1,400
		≥60	258	284	730	424	1,168
	Female	18–44	338	258	862	368	1,144
		45–59	615	310	861	352	1,242
		≥60	239	305	825	368	1,152
Non-dominant gastrocnemius	Male	18–44	277	157	848	269	1,298
		45–59	339	257	856	304	1,272
		≥60	280	215	767	304	1,312
	female	18–44	337	160	816	447	1,081
		45–59	619	293	843	400	1,376
		≥60	240	136	812	337	1,152
Dominant gastrocnemius	Male	18–44	280	192	759	208	1,136
		45–59	319	212	847	304	1,312
		≥60	260	231	804	328	1,487
	Female	18–44	335	153	811	262	1,178
		45–59	611	258	861	357	1,403
		≥60	240	251	951	304	1,151
Non-dominant vastus medialis	Male	18–44	279	227	852	448	1,728
		45–59	339	190	822	304	1,312
		≥60	240	132	770	352	1,328
	Female	18–44	338	142	837	304	1,136
		45–59	612	255	823	405	1,323
		≥60	238	262	872	336	1,072
Dominant vastus medialis	Male	18–44	277	175	792	383	1,378
		45–59	340	197	816	408	1,248
		≥60	277	132	677	319	1,152
	Female	18–44	340	102	646	304	1,215
		45–59	615	251	814	374	1,232
		≥60	240	210	745	336	1,264
Non-dominant SCM	Male	18–44	298	260	776	240	800
		45–59	339	197	971	240	928
		≥60	299	254	916	256	840
	Female	18–44	320	215	801	208	848
		45–59	618	316	1,027	304	968
		≥60	220	235	881	264	640
Dominant SCM	Male	18–44	299	344	1,014	304	888
		45–59	337	127	979	192	866
		≥60	300	279	932	240	912
	Female	18–44	339	204	926	192	824
		45–59	620	340	1,182	304	1,032
		≥60	220	249	871	192	704

ADM, abductor digiti minimi; EDC, extensor digitorum communis; SCM, sternocleidomastoid.

### The judgment criteria

**Figure 5:**
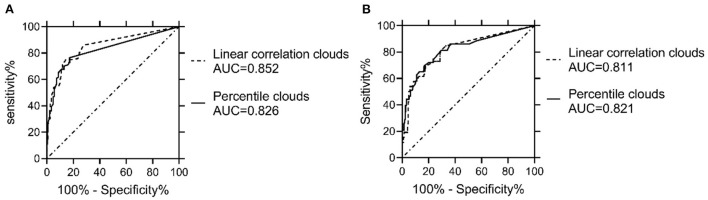
Comparison of ROCs and AUC between the linear regression clouds and percentile clouds. The ROCs were drawn with the percentage of data points beyond the boundaries of the linear regression clouds and percentile clouds, The AUC of the linear regression clouds and percentile clouds were both >0.8. AUC = area under the curve. **(A)** The AUC of both the linear regression clouds and percentile clouds that were used to discriminate neuropathic vs. non-neuropathic lesion were >0.8. **(B)** The AUC of both the linear regression clouds and percentile clouds that were used to discriminate myopathic vs. non-myopathic lesion were >0.8.

**Figure 6:**
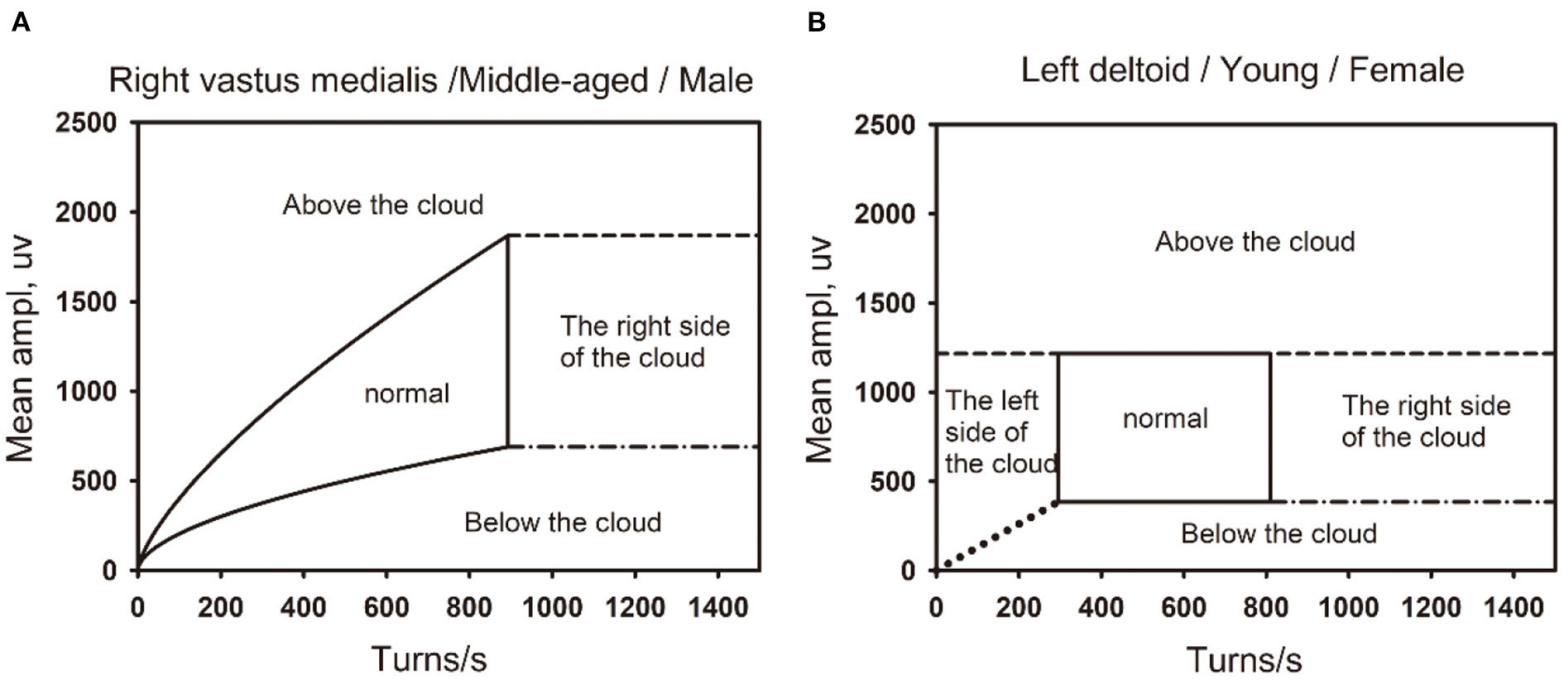
The boundaries of the the linear regression clouds and percentile clouds are shown in the figure. **(A)** The normal linear regression clouds and its boundary. The right vastus medialis muscle in a middle-aged (45–59) male. **(B)** The normal percentile cloud and its boundary. The left deltoid muscle in a Young-aged (18–44) female.

**Figure 7:**
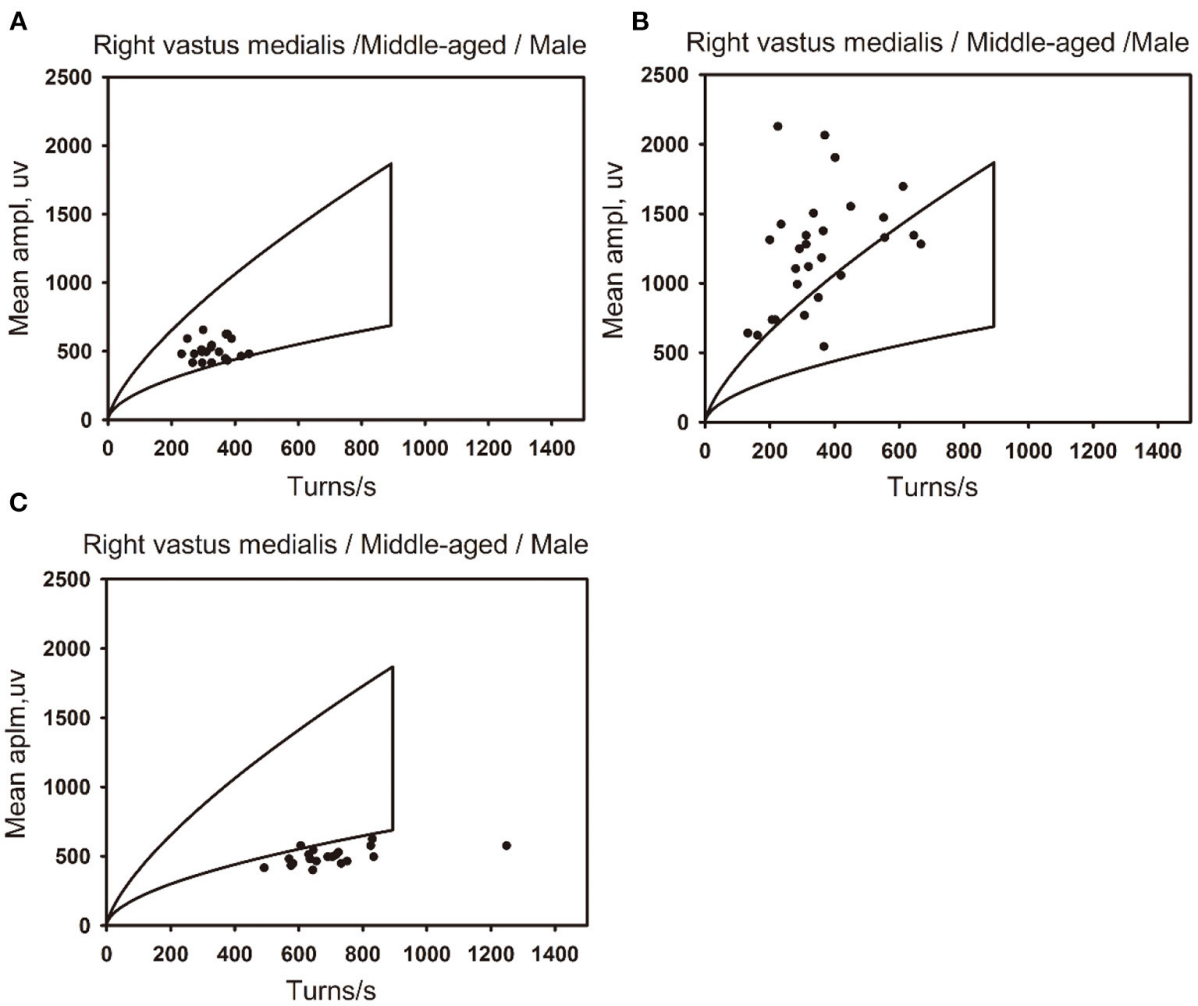
The distribution of data points for normal, neuropathic lesion, and myopathic lesion muscles in the linear regression clouds. The linear regression clouds of the right vastus medialis muscle in three middle-aged men (44–59 years old). **(A)** The right vastus medialis muscle of a middle-aged man is defined as normal, with the muscle stats distributed within the linear regression cloud. **(B)** The right vastus medialis muscle of a middle-aged man is defined as neuropathic lesion, with the data points of muscle beyond the upper boundary of the linear regression cloud, which exceed 25% of all data points. **(C)** The right vastus medialis muscle of a middle-aged man is defined as myopathic lesion, with the data points of muscle beyond the lower and right boundaries of the linear regression cloud, which exceed 18% of all data points..

**Figure 8:**
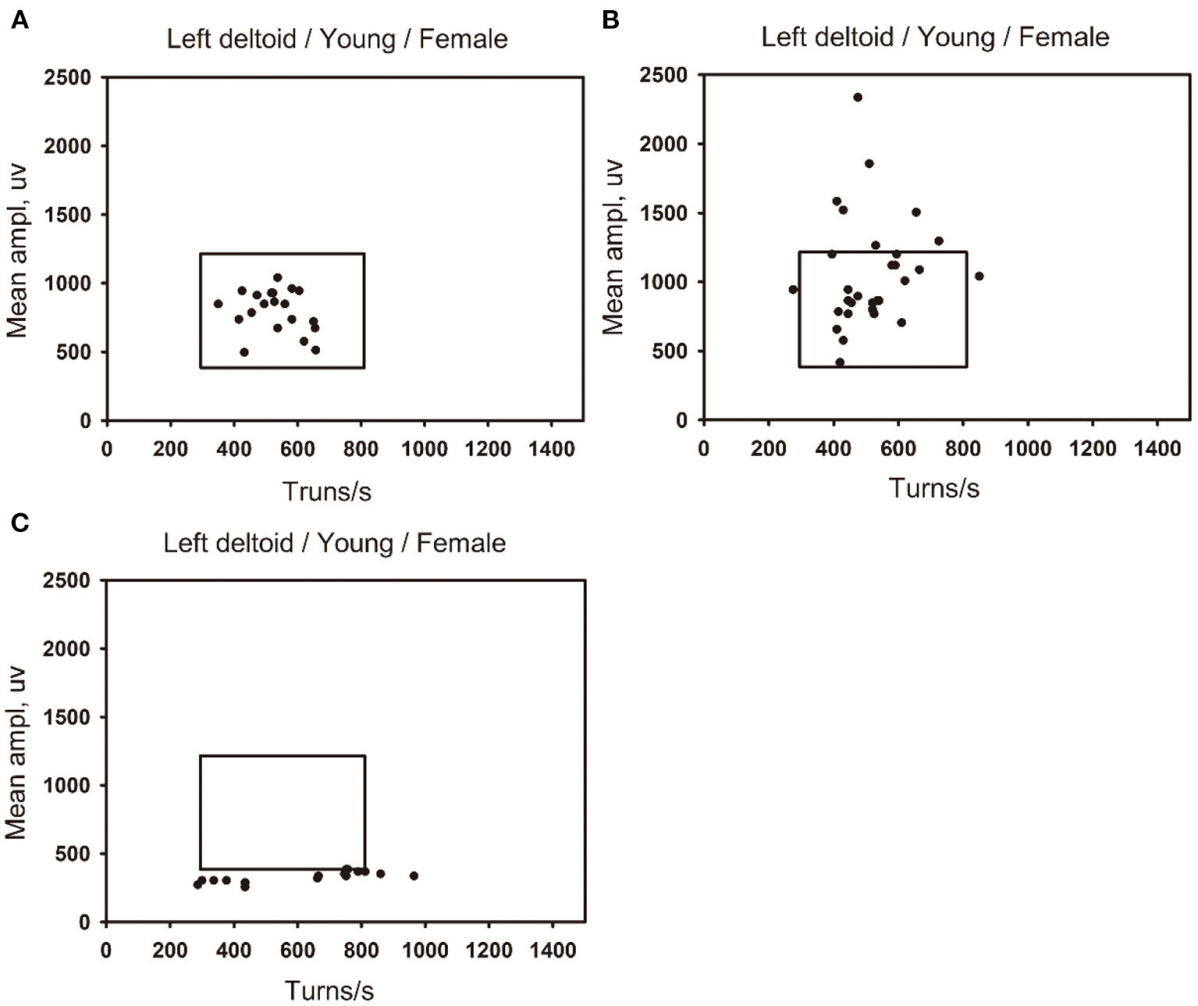
The distribution of data points for normal, neuropathic lesion, and myopathic lesion muscles in the percentile clouds. The percentile clouds of left deltoid muscle in three young women (18–44 years old). **(A)** The left deltoid muscle of a young women is defined as normal, with the muscles data points distributed within the linear regression cloud. **(B)** The left deltoid muscle of a young women is defined as neuropathic lesion, with the data points of muscle beyond the upper boundary of the percentile cloud, which exceed 20% of all data points. **(C)** The left deltoid muscle of a young women is defined as myopathic lesion, with the data points of muscle beyond the lower and right boundaries of the percentile cloud, which exceed 30% of all data points.

The ROCs were drawn with the percentage of data points beyond the boundaries of the linear regression clouds and percentile clouds, respectively (see Figure 5). The sensitivity and specificity corresponding to the percentage calculated through the data point of normal muscle, neuropathic and myopathic lesions muscle beyond the boundaries of clouds are shown in Tables 9–11. With priority of specificity for determining criteria, according to ROC, the cut-off value corresponding to the specificity > 94% was taken as a criterion which discriminate between neuropathic lesion vs. non-neuropathic lesion, myopathic lesion vs. non-myopathic lesion, and abnormal muscles vs. normal muscles (see Tables 9–11, Figure 6). For the linear regression clouds, if over 25% of the data points exceed the upper boundaries, neuropathic lesion was determined; if over 18% of the data points exceed the lower and right boundaries, myopathic lesion was determined (see Figure 7). For the percentile clouds, if over 20% of the data points exceed the upper boundaries, neuropathic lesion was determined; if over 30% of the data points exceed the lower and right boundaries, myopathic lesion was determined (see Figure 8). When the data points exceed the upper boundaries and the lower-right boundaries simultaneously, the determination was made according to whether the difference between the data points beyond the bilateral boundaries exceeds the determining criteria. The AUC of the linear regression clouds and percentile clouds were both >0.8, revealing that both clouds have high diagnostic values (see Figure 5, Tables 9, 10).

**Table 9:** The sensitivity and specificity of each method for discrimination of neuropathic vs. non-neuropathic lesion muscles.

	**The percentage beyond the upper boundary of the clouds**	**AUC**	**Youden's index**	**Sensitivity**	**Specificity**	***P* of sensitivity comparison of the three methods**	***P* of specificity comparison of the three methods**
QMUP			0.4145	47.46%	93.99%		
Linear regression clouds	>25%	0.852	0.4424	48.44%	95.80%		
Percentile clouds	>20%	0.826	0.4659	52.17%	94.42%		
						0.521	0.767

AUC, Area under the curve; QMUP, Quantitative assessment of the motor unit potential.

**Table 10:** The sensitivity and specificity of each method for discrimination of myopathic vs. non-myopathic lesion muscles.

	**The percentage beyond the lower-right boundary of the clouds**	**AUC**	**Youden's index**	**Sensitivity**	**Specificity**	***P* of sensitivity comparison of the three methods**	***P* of specificity comparison of the three methods**
QMUP			0.2297	27.91%	95.06%		
Linear regression clouds	>18%	0.811	0.3643	42.31%	94.12%		
Percentile clouds	>30%	0.821	0.4115	46.51%	94.64%		
						0.186	0.871

AUC, area under the curve; QMUP, Quantitative assessment of the motor unit potential.

**Table 11:** The sensitivity and specificity of each method for discrimination of abnormal vs. normal muscles.

	**The percentage beyond the upper boundary of the clouds**	**The percentage beyond the lower-right boundary of the clouds**	**Youden's index**	**Sensitivity**	**Specificity**	***P* of sensitivity comparison of the three methods**	***P* of specificity comparison of the three methods**
QMUP			0.2536	44.83%	80.53%		
Linear regression clouds	>25%	>18%	0.3322	49.35%	83.87%		
Percentile clouds	>20%	>30%	0.3615	51.41%	84.74%		
						0.241	0.531

QMUP, Quantitative assessment of the motor unit potential.

### Sensitivity and specificity

In a word, QMUP, the linear regression clouds, and percentile clouds can accurately discriminate the neuropathic or non-neuropathic lesion, the myopathic or non-myopathic lesion. It should be noted that the specificities of QMUP, the linear regression clouds, and percentile clouds were basically identical to each other, whereas the sensitivity and Youden′s index of the percentile clouds were higher than those of QMUP and the linear regression clouds (Tables 9, 10). These findings revealed that, with the same specificity, the true positive rate of the percentile clouds was the highest and the percentile clouds had thus significant diagnostic values.

## Discussion

There are mainly three limitations in the application of QMUP. First, the measurement of duration of MUP through QMUP depended largely on the subjective judgment of the EMG technician (3), and the sampling of MUP are subjective. As a result, sampling bias can be caused easily (1). Furthermore, QMUP only analyzes the motor units activated by small force contractions, and it can only find motor unit damage with a lower excitement threshold (3). Therefore, QMUP cannot reflect the overall situation of all the motor units. If the motor units with a higher excitement threshold were affected at an earlier stage of the disease and the reduction of motor unit amplitude appear before the duration reduction in myopathic lesion, the duration of motor unit potential did not change, therefore, abnormalities cannot be detected by QMUP (1). Finally, the QMUP examination takes a long time, and some patients cannot complete it due to pain (13).

### Development of automatic analysis technology of EMG interference pattern

With the increase of contraction force, many motor units were excited simultaneously, forming interference patterns. In the past, the visual assessment of interference patterns was often used, which was qualitative and subjective (1). To solve these problems, automatic analysis technology of EMG interference pattern was developed.

In 1964, Willison reported a technique to measure the turn of various activated motor units for the first time (14). In 1967, Rose and Willison first analyzed the number of turns per second and the mean amplitude of EMG under standard contraction force (15). In 1975, Fuglsang-Frederiksen and Månsson exploited the maximum contraction force of a single subject to measure the turn and mean amplitude (16). In 1983, Stålberg et al. found that for different muscles, there were different linear correlations between the number of turns per second and the mean amplitude. Also, the number of turns per second and the mean amplitude of most muscles were linearly positively correlated, but those of the vastus medialis were not linearly correlation (4). Following the same technique, in 1991, Nandedkar et al. (5) found that the cloud of the tibialis anterior was similar to that obtained by Stålberg, while the cloud of biceps brachii was quite different.

The shape and reference values of the turn-amplitude clouds obtained by different researchers were different (4–7). Nandedkar et al. (5) and Jabre et al. (6) believed that the differences in the shape of the clouds were caused by the differences in the contraction force of the examinees when the data were collected. Also, the shape of the clouds largely depended on whether the data were collected in the maximum contraction force (6).

Nandedkaret et al. (5) used a dynamometer to collect data at 10–80% of the maximum contraction force. It was found that when starting from a small contraction force to 50% of the maximum contraction force, the increase of the number of turns per second was greater than that of the mean amplitude, while the increase of the mean amplitude was greater than that of the number of turns per second at a higher contraction force. Jabre et al. (6) found that for a small to moderate contraction force, the number of turns per second and the mean amplitude were linearly correlated, with a small standard deviation. When the contraction force was increased from 50% of the maximum contraction force, the increase in mean amplitude was more significant than that in the number of turns per second. There was no linear correlation between the number of turns per second and the mean amplitude, and the standard deviation became larger. The Stålberg linear regression method was not suitable for analyzing the data collected from a small contraction force to the maximum contraction force.

In our study, we used linear regression and percentile method to establish two kinds of clouds. This study analyzed the factors that affect the number of turns per second and the mean amplitude of electromyographic interference patterns, including gender, age, and dominant and non-dominant limb muscles. For all the muscles, the reference values of the percentile clouds for different genders, ages, and dominant and non-dominant side of the muscles of same name have been established. For the muscles that can adopt the linear regression clouds, the reference values of the linear regression clouds were established for different groups according to gender, age, and dominant and non-dominant limb muscles of same name. The common characteristics of the two clouds were as follows: Firstly, the data were collected from a small force to the maximum contraction force, which included most of the motor unit information. Secondly, in this study, the threshold of 100 μV was not subtracted from the mean amplitude, and the data points with the mean amplitude of less than 100 μV were deleted after data collection. Based on this, the obtained the clouds were closer to the true distribution of the data points. Previous studies of turn-amplitude clouds used mean amplitude minus the threshold of 100 μV to draw the clouds (1, 4, 6, 7). Thirdly, the grouping was more complete, and the reference values were established according to gender, age, and dominant and non-dominant limb muscles of same name. Finally, it was reproducible and intuitive. It can present the position of each data record in the clouds during the examination, enabling quick judgments and shortening the examination time to reduce pains.

### The feature of the linear regression clouds

The turn-amplitude clouds drawn in this study differ from previous clouds in three aspects. First, there was no linear correlation between the number of turns per second and the mean amplitude of some muscles, and the linear correlation method cannot be applied to draw the clouds. Unless a dynamometer was used, it was difficult to accurately measure the contraction force in actual work. As a result, for some muscles, there does not exist a linear correlation between the number of turns per second and the mean amplitude collected. Second, takes the standard errors of the intercept and slope of the linear regression equation and substitute they into the linear regression equation as the upper and lower boundaries of the clouds, including the prediction interval of the mean amplitude corresponding to the given number of turns per second. The methods to draw turn-amplitude clouds previously (1, 4, 6, 7) all take intercept ± 2SD of the linear regression equation as the upper and lower boundaries of the clouds, including the 95% confidence interval of the mean amplitude corresponding to a given number of turns per second. Third, also, the right boundary takes 99 percentiles, and the left boundary takes the minimum value of the number of turns per second of the muscle. The previous developing methods of the turn-amplitude clouds all adopted the 99 percentile of the number of turns per second as the right boundaries of the clouds (1, 4, 6, 7). The definitions of the high boundaries were different in previous studies.

The application of the linear regression clouds has its limitations. That means, it can be applied only when there is linear correlation between the number of turns per second and the mean amplitude with normal distribution and the linear regression equation can be set up. The linear regression clouds cannot be applied to all muscles, we thus developed the percentile clouds.

### The feature of the percentile clouds

By contrast, percentile clouds have the following advantages. Firstly, it did not require the data to be normally distributed, and it was applicable to all the muscles. Secondly, the percentile clouds collect data from a small contraction force to the maximum contraction force, which can more comprehensively reflect the motor unit information. Notice that it was impossible to use a dynamometer to measure contraction force in clinical practice, therefore examiners can hardly determine the contraction force accurately. The percentile clouds have no precise requirements for contraction force, thus it can be extensively used in automatic EMG interference pattern analysis. Thirdly, the sampling bias was smaller because it does not rely on the subjective judgment of the examiners, and the subjects can cooperate to complete data collection. Additionally, there was no statistical difference in the specificity of the linear regression clouds, the percentile clouds, and QUMP, but the sensitivity and Youden's index of the percentile clouds were the highest and had significant diagnostic value. Therefore, the percentile clouds were recommended to apply for automatic analysis of electromyography interference pattern.

### The evidence and advantage of judgment criteria

Our study is the first to determine criteria based on ROCs. The previous studies of the turn-amplitude clouds adopted different judgment criteria that is the percentage of the data records beyond the boundaries of the clouds, the evidence of which had not been explained (1, 4, 6, 7). The EMG is a diagnostic examination. Due to its long examination time and great pain on the subject, it is not suitable for screening of disease. Therefore, the determination of determining criteria should be given priority to the improvement of specificity, so that the false positive rate is as low as possible. The judgment criteria with a higher specificity usually have a lower sensitivity. The low sensitivity corresponds to the low true positive rate, namely, the high false negative rate. As a result, the false negatives cannot be excluded from a normal result of EMG. In this study, according to the principle of giving priority to specificity and referring to ROCs, the cut-off value with specificity >94% was selected as the judgment criteria for distinguishing neuropathic lesion vs. non-neuropathic lesion and myopathic lesion vs. non-myopathic lesion in both clouds, which is statistically reasonable. Since the criteria have taken into account the specificity of the EMG examination (namely, the consumption of examination time, pain for the subjects, and requiring Subjects' cooperation), they are suitable for diagnostic examination. Since the sensitivity and specificity of the two clouds are different, the judgment criteria of the two clouds are different, namely, the percentages beyond the boundaries of the two clouds are different when the judgment criteria with the specificity >94% are adopted for both clouds. The AUC of both the linear regression clouds and percentile clouds are >0.8, indicating that both clouds have high authenticity and diagnostic values.

### Analysis of sensitivity and specificity

The percentile clouds possess higher sensitivity and authenticity. With clinical diagnosis as the gold standard, this study compared the sensitivity and specificity of the linear regression clouds, percentile clouds, and QMUP, and there is no significant statistical difference in the specificity of the three methods, but the sensitivity and Youden's index of the percentile clouds are the highest, indicating that there is significant diagnosis value for the percentile clouds. On the premise of similar specificity, the Youden's index of the percentile clouds is higher than that of the linear regression clouds, indicating that the percentile clouds possess higher authenticity and stronger ability to discriminate neuropathic lesion vs. non-neuropathic lesion and myopathic lesion vs. non-myopathic lesion. Nirkko et al. (1) reported that the turn-amplitude cloud had the same specificity as QMUP, but its sensitivity was higher than that of QMUP. Peng et al. (12) demonstrated that the turn-amplitude clouds had higher sensitivity for discriminating myopathic and non-myopathic lesion than QMUP and there was no statistical difference in specificity; there was no statistical difference in the sensitivity and specificity of the turn-amplitude clouds and QMUP for discriminating neuropathic vs. non-neuropathic lesion and normal vs. abnormal.

This study also has its limitations. Above all, the analysis of sensitivity and specificity adopts clinical diagnosis as the gold standard, so it is not independent. Also, the results of QMUP are taken into account in the clinical diagnosis, which may overestimate the sensitivity and specificity of QMUP (1, 14). Besides, the sample size is small, because the EMG examination is painful for subjects, which makes it difficult to recruit volunteers.

## Conclusions

In this work, we study reference values of the linear regression clouds and percentile clouds, and compare the sensitivity and specificity of both clouds and QMUP, and we have developed a new judgment criterion. First, we analyze the factors that affect the number of turns per second and the mean amplitude of the EMG interference pattern. Second, the linear regression method and the percentile method are exploited to draw the turn-amplitude clouds and establish their reference values. Third, it is more rational to determine their judgment criteria respectively according to the ROCs of the two clouds. Compared with the specificity of the linear regression cloud, the percentile cloud, and QUMP, there is no difference, but the sensitivity and Youden's index of the percentile clouds are highest and have higher diagnostic value. Fourth, it is more reasonable for the upper and lower boundaries of the linear regression clouds to include the prediction interval. However, the linear regression cloud cannot be applied to all muscles. Fifth, it is easy for subjects to cooperate that inspection process of the percentile clouds has no precise requirements for contraction force when the data collection is performed from small to the maximum contraction force. Besides, it is faster than QMUP and has no selection bias. Finally, the percentile clouds have no special requirements that the number of turns per second and the mean amplitude have normal distribution, consequently it has a wider range of applications than the linear regression clouds. Therefore, the percentile clouds can be adopted as the optimum for automatic electromyography interference pattern analysis.

## Data availability statement

The original contributions presented in the study are included in the article/Supplementary material, further inquiries can be directed to the corresponding author/s.

## Ethics statement

This study was approved by Ethics Committee of First Affiliated Hospital of Kunming Medical University of China. The patients/participants provided their written informed consent to participate in this study. Written informed consent was obtained from the individual(s) for the publication of any potentially identifiable images or data included in this article.

## Author contributions

DW: conception of the work and writing articles. ZL: conception of the work and recruit volunteer. LL and RX: recruitment of volunteer and data acquisition. YG, TW, and YK: data collation and data preservation. JW, QY, and NL: statistical analysis. YH: conception of the work, supervision, and manuscript revision. All authors contributed to the article and approved the submitted version.

## Funding

This study was supported by Yunnan Science and Technology Department—Kunming Medical University Application Basic Research Joint Special Fund Project [Grant No. 2018FE001(-207)], Yunnan health training project of high level talents (L-2019019), Yunnan High-level Talent Training Support Program Famous Doctor Special project (RLMY20200005), The Major Science and Technology Special Project of Yunnan Province (202102AA100061), and Yunnan Science and Technology Department—Kunming Medical University Application Basic Research Joint Special Fund Project [Grant No. 2019FE001(-208)].

## Conflict of interest

The authors declare that the research was conducted in the absence of any commercial or financial relationships that could be construed as a potential conflict of interest.

## Publisher's note

All claims expressed in this article are solely those of the authors and do not necessarily represent those of their affiliated organizations, or those of the publisher, the editors and the reviewers. Any product that may be evaluated in this article, or claim that may be made by its manufacturer, is not guaranteed or endorsed by the publisher.

## Abbreviations

AUC: area under the curve
EMG: Electromyography
MUP: motor unit potential
QMUP: Quantitative assessment of the motor unit potential
ROC: receiver operator characteristic curve
SD: standard deviation.
