# Supplementary material for: On the establishment of reference values of clouds of electromyography interference pattern by linear regression method and percentile method and comparison of sensitivity and specificity of both methods
Source: Front Neurol. 2022 Sep 1;13:917308. doi: 10.3389/fneur.2022.917308 (PMC9475198; doi:10.3389/fneur.2022.917308)
Supplement: Supplementary file 1 [file Presentation_1.pdf]

Right gastrocnemius of older female

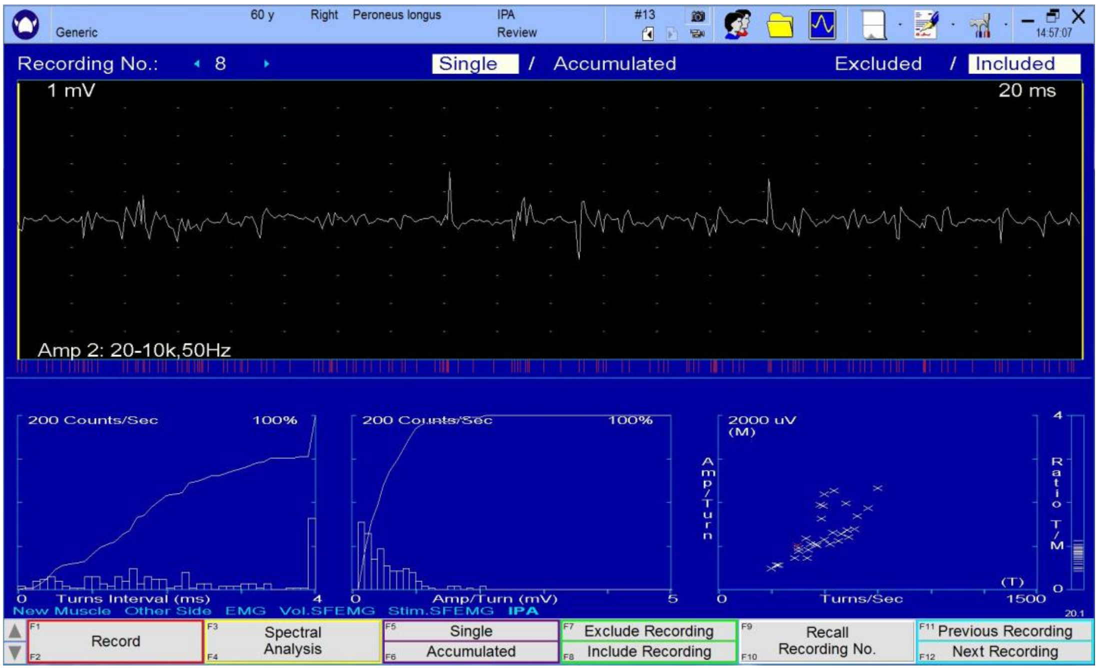

Figure_s1: The shape and frequency of the motor unit on the display screen of the electromyography for small force contraction. It shows motor units with the lower number of turns per second and the mean amplitude.

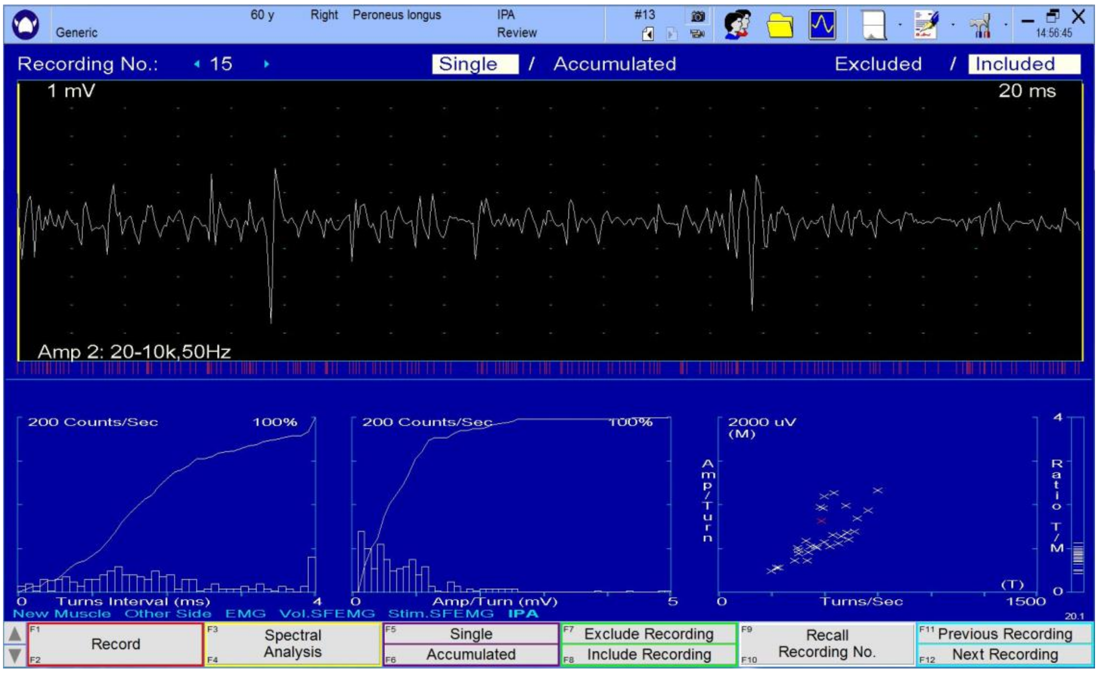

Figure_s2: The shape and frequency of the motor unit on the display screen of the electromyography for moderate force contraction. It shows that with the increasing of the number of turns per second of the motor unit, the mean amplitude of the motor unit increases gradually.

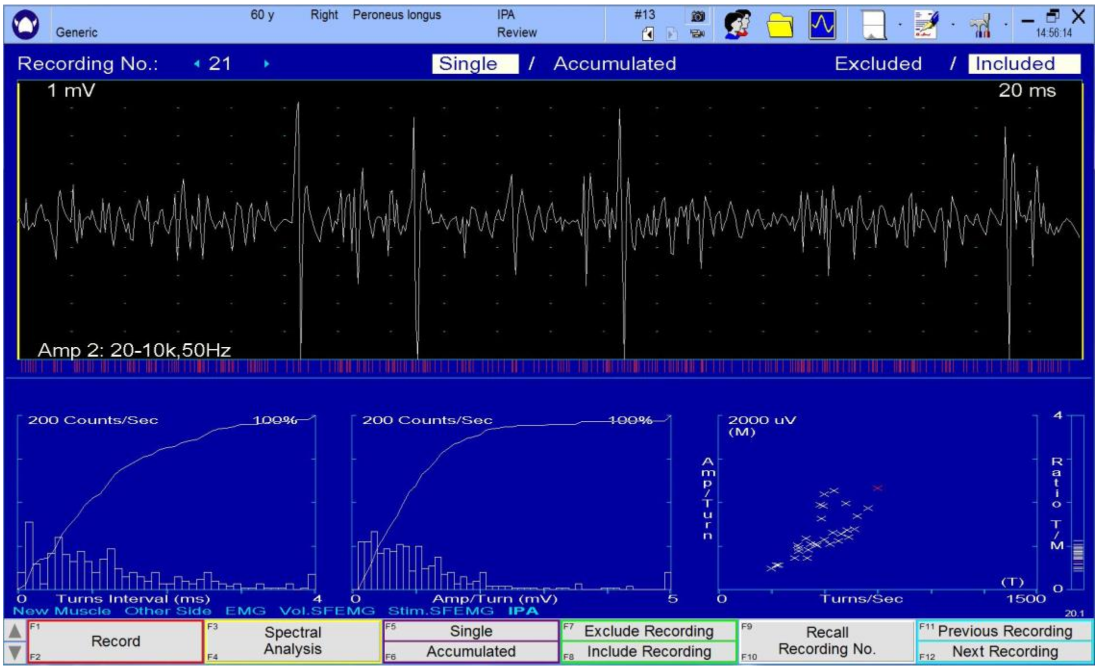

Figure_s3: The shape and frequency of the motor unit on the display screen of the electromyography for maximal force contraction. It shows that when the number of turns per second of the motor unit reaches the maximum, its mean amplitude also reaches the maximum.
